# Beliefs, referrals, and mental healthcare pathways in the Eastern Democratic Republic of Congo

**DOI:** 10.1371/journal.pgph.0006715

**Published:** 2026-07-02

**Authors:** Martial Mumbere Vagheni, Jean-Bosco Kahindo Mbeva, Astride Lina Piripiri, Joseph Zawadi Kavulivwa, Rosemary Ricciardelli, Daniel Okitundu Luwa E Andjafono, Bives Mutume Nzanzu Vivalya

**Affiliations:** 1 Department of Mental Health, Université pour la Conservation de la Nature et le Développement de Kasugho, Goma, Democratic Republic of the Congo; 2 Department of Psychiatry, University of Kinshasa, Lemba, Kinshasa, Democratic Republic of the Congo; 3 Department of Internal Medicine, Katwa General Referral Hospital, Butembo, Democratic Republic of the Congo; 4 Department of Health Sciences, Official University of Rwenzori, Butembo, Democratic Republic of the Congo; 5 Kinshasa School of Public Health, University of Kinshasa, Kinshasa, Democratic Republic of the Congo; 6 Department of Psychiatry, Kampala International University Western Campus, Bushenyi, Uganda; 7 Department of Sociology, Memorial University of Newfoundland, St. John’s, Newfoundland and Labrador, Canada; PLOS: Public Library of Science, UNITED STATES OF AMERICA

## Abstract

Although the primary healthcare (PHC) system gatekeeps early access to mental healthcare services, little is known about the factors influencing the use of these services by people with mental disorders living in conflict zones of Eastern Democratic Republic of Congo. The current study describes the patterns and factors associated with pathways to mental healthcare, with an emphasis on how religious beliefs and referral patterns are associated with the use of PHC. We surveyed patients (n = 404) attending nine psychiatric hospitals to elucidate the pathway used for those with mental health needs to obtain care. Binary logistic regressions were performed to identify factors associated with first, second, third, and fourth points of contact when seeking care. In total, 47.3% of patients had their first care contact at a psychiatric hospital while 89.3% were self-referred. Roughly one-third of participants’ first point of contact was a religious leader. Asked about sources of their compromised mental health, 32.7% of respondents reported witchcraft, 30% supernatural powers, and 12.5% divine punishment. Having a family history of mental disorders and a religious affiliation other than being Pentecostal and non-Pentecostal (aOR=0.17, p = 0.026) were associated with lower odds of non-PHC first contact (aOR=0.06, p = 0.004). Additionally, having multiple psychiatric episodes (aOR=9.86, p = 0.028), self-referral (aOR=6.37, p < 0.001), and attributing challenges to divine punishment (aOR=4.68, p = 0.05) or witchcraft (aOR=2.35, p = 0.04) were associated with higher odds of non-PHC first contact. Findings reveal the significant underutilization of PHC for mental health needs in conflict zones, individuals instead favoring self-referral to psychiatric hospitals or religious leaders. This behavior is driven by cultural and religious beliefs, specifically attributing mental disorders to witchcraft, divine punishment, and lack of integrated mental health services within the PHC system. We conclude with discussion of a collaborative model between religious leaders and medical professionals to improve care pathways in conflict zones.

## Introduction

In low-income countries (and high-income countries too), many individuals with mental health needs are treated in primary healthcare (PHC) hospitals, which serve as gatekeepers to psychiatric hospitals [1]. The PHC facilities are the first point of contact for those with health needs, because their community health workers and general practitioners provide accessible and comprehensive care, before referring patients in need to specialized settings, like psychiatric hospitals. Efforts to integrate mental healthcare into the PHC system are affected by factors like low mental health prioritization, a shortage of psychotropic medications, and the scarcity of specialists at government hospitals [2].

Cultural myths and religious beliefs also influence how individuals with mental health needs interpret and seek help [3,4], especially from religious leaders and traditional healers [5]. Yet, scholars have not focused on the role of religious beliefs on mental healthcare pathways in conflict-affected regions. An individual’s socio-economic status, belief in the role of supernatural powers in disease etiology [6], and availability of specialized mental healthcare services influence people’s attitudes towards the management of psychiatric disorders [7–10]. These factors negatively affect treatment seeking behaviors, especially in conflict zones, where one in five individuals suffer from at least one mental health disorder [11].

Political conflicts and civil unrest have affected the Eastern region of Democratic Republic of Congo (DRC) for more than two decades. Here, six in ten individuals who attend religious centers for spiritual assistance meet the diagnostic criteria of at least one psychiatric disorder [12]. Seven in ten people attend psychiatric hospitals only after seeking help from traditional healers or religious leaders [5]. While 20% to 50% of the population may have post-traumatic stress disorder (PTSD) or depressive symptoms due to combat exposure [13,14], only a tiny fraction (1.9% to 2.5%) of these cases is recorded or identified within the PHC facilities [12]. In addition, people with mental health needs use several pathways to care, including self-referral [3,10,15]. Barriers to access to mental healthcare services in conflict zones stem from poor integration of mental health in the PHC system to inadequate understanding of the burden of psychiatric disorders. This is particularly the case in jurisdictions of war, poverty, low education, and loss of property. However, contextual factors limiting the use of PHC by individuals with mental health challenges have not yet been deeply assessed in conflict zones.

The Cultural Determinants of the Help Seeking Model (CDHSM) provides an avenue to understand how culture impedes help-seeking behavior for those with compromised mental health. The model includes facilitators and barriers to mental health service provision, such as causal attributions, social significance, context dynamics, and resource availability [4]. We used the CDHSM to frame the current study as it underpins the role of cultural and religious factors in mental health help-seeking behavior. Further, we reflected on the World Health Organization’s Mental Health Gap Action Program [16–18], which is not fully integrated in Eastern DRC. Overall, we aimed to describe the patterns and factors associated with pathways to mental healthcare, with an emphasis on how religious beliefs and referral patterns are associated with the use of PHC.

## Method

### Study design and setting

We conducted a cross-sectional survey involving patients who attended nine psychiatric hospitals in war torn Eastern DRC between 15^th^ August and 15^th^ December 2021. The selected hospitals included: Polycliniques Sainte Croix of Mulo, Cap Salama, Centre Muyisa, Centre Diaconal Dr. Rohland (Cediar), Bora Uzima, Notre Dame de Lourde, Centre La Guérison, Centre de Relance en Santé Mentale, and Centre pour la Protection des Indigents et de Malades Mentaux (CEPIMA). These facilities met inclusion criteria of: (i) being in a conflict-affected region of North-Kivu, Eastern DRC, (ii) belonging to private managers, and (iii) providing biological and psychosocial therapies to individuals with mental disorders for at least two years and (iv) identified in respective health zones as specialized psychiatric hospitals that receive referral from PHC system. These hospitals have an average bed-capacity of 50. The common mental health disorders of patients in these psychiatric hospitals are substance-use related problems, bipolar affective disorder, schizophrenia spectrum disorders, epilepsy, and major depressive disorder.

### Study participants

Participants were patients admitted to psychiatric hospitals, aged between 16 and 65 years, consented to participate by providing an informed written consent or assent, and met the fifth edition of the Diagnostic and Statistical Manual of Mental Disorders (DSM-V) criteria for substance use disorder, bipolar affective disorder, schizophrenia spectrum disorders, major depressive disorder, and posttraumatic stress disorder. Exclusion criteria were patients with cognitive impairment who would impair their ability to comprehend the consent form and the questionnaire. The sample size of 404 participants was determined using the modified Daniel’s formula for cross-sectional studies [19] with a population proportion of 50%, the 95% confidence level, and 5% margin of error. Participants with missing responses were excluded from the analysis, except for the question about religious beliefs and referral patterns, where five respondents did not answer. No data imputation was performed. The heterogenic distribution of the sample across the nine hospitals is presented in (S1 Table).

### Data collection, instruments, and variables

With the permission of the executive directors, we reviewed the medical records of potential participants on arrival, then obtained psychiatric diagnoses. We utilized consecutive sampling techniques during the recruitment process going from 15^th^ of August to 15^th^ of December in 2021. Trained research assistants used Kobo Collect to administer a structured survey to eligible patients who provided written informed consent or assent. A structured questionnaire, developed for this study, was piloted among 40 participants and revised based on pilot data feedback. The questionnaire was translated from French to Kiswahili by two professional translators with a mental health background. To maintain inter-rater reliability and data quality, all research assistants received standardized training on survey administration, ethical conduct, and use of the digital data collection tool. Field supervision was conducted throughout data collection. Here, we conducted periodic joint interviews, and daily consistency checks of uploaded forms to identify and resolve discrepancies in real time.

We administered face-to-face surveys in person, each 45 and 60 minutes in duration. Collected socio-demographic information included age, sex, marital status, level of education, occupation, and religion. Clinical data included information about patterns of admissions: place (i.e., referral hospital), mode of admission (referred versus self-referred), type (new admissions and readmissions) and number of admissions. We also collected information on the age of onset of psychiatric symptoms, evolution of the psychiatric diagnosis starting from the first episode of presentation, recurrent acute episodes, progressive disease or unknown evolution mode; and family history of mental disorders.

Regarding religious orientation, we collected information by asking: “What is your religion?” with answer options of “I am Catholic, Protestant, Adventist, Muslim, or other”. We then asked about religiosity: ‘How would you categorize yourself as religious? Religiosity was categorized based on the frequency of attendance at religious services, an approach commonly applied in major international surveys [20,21], and responses from pilot testing. In line with these instruments, we defined three levels: *very religious* (more than once per week), *moderately religious* (weekly to a few times per month), and *indifferent* (less than once per month or never). We also assessed how participants interpreted their mental health challenges, asking: “In your opinion, what is the cause of the mental disorder?” with answer options of “natural disease, Divine punishment, witchcraft, other mentioned causes, and unknown causes.”

We refer to care pathways as the avenue pursued by any individual with compromised mental health to reach the appropriate mental health treatment center [9]. To measure pathways to mental healthcare, we used an adapted version of the collaborative World Health Organization’s “Pathway Study” encounter form. The form outlines care-seeking behaviors and treatment pathways known to be used by individuals with mental health challenges before they seek healthcare in psychiatric hospitals [15]. The WHO encounter form is 22 item semi-structured questionnaire that records the patient’s “pathway contacts” referring to who the patient initially sought treatment from, followed by second, third, etc. treatment sought, while including information on delays, and referral sources. Traditional healers and faith healers are examples of informal providers of treatment used in low- and middle-income countries to map help-seeking behavior, identify delays in receiving care, and compare healers to professional health facilities. In certain contexts (like Ethiopia), the pathway has been characterized as both a possible and acceptable method for gathering pertinent pathway data [22]. The mental health care referral pathway was assessed by recording the sequence of providers consulted by each participant before reaching psychiatric services. Participants were asked to identify their first, second, third, and fourth or subsequent point of contact for mental health care. For each step, the type of provider consulted was categorized as community health worker, religious leader, traditional healer, primary health care facility, psychiatric hospital, or any other unidentified provider. Then, we grouped participants in two categories for each contact point: PHC workers (i.e., community health workers, and medical professionals in health centers and general hospital), and all other stakeholders (i.e., psychiatric nurses, clinical psychologists, religious leaders, traditional healers, and unidentified actors) as non-PHC workers.

### Data processing and analysis plan

Statistical analyses were performed using the R Studio Integrated Development Environment Version R 4.2.2. Using the (S1 Data), we summarized descriptive statistics as absolute frequencies and percentages for categorical variables, means, and standard deviations or medians and interquartile ranges for continuous variables. Comparisons between demographic and clinical factors were examined using a chi-squared test for categorical variables (regarding the different points of contact for care). We conducted separate binary logistic regression analyses for each stage of care-seeking (first, second, third, and fourth point of contact) to identify factors associated with the use of non-primary healthcare (non-PHC) services versus PHC service at each stage. For each model, the dependent variable was coded as 1 = non-PHC contact (e.g., religious leaders, traditional healers, psychiatric hospitals) and 0 = PHC contact. Analyses at each stage were restricted to participants who had reached that stage in their care pathway (404 participants at the first contact, 308 for the second, 179 for the third and 104 for the fourth or more contact). Because participants were recruited from multiple psychiatric hospitals, cluster-robust standard errors at the hospital level were used to account for within-hospital correlation. Predictor variables included sociodemographic characteristics (age, sex, marital status, employment), cultural and belief-related factors (religious affiliation, religious involvement, causal attribution), and clinical characteristics (admission mode, illness severity, recurrence, and family history of mental disorders) based on existing studies [23–25]. Factors associated with the outcome at p < 0.20 in bivariate analyses were entered into multivariate models. Adjusted odds ratios (aOR) with 95% confidence intervals were reported, and statistical significance was set at p < 0.05. These models were estimated separately for each contact stage because determinants of initial help-seeking may differ from factors influencing subsequent care decisions.

### Ethical considerations and reporting guidelines

Ethical approval was provided by the Ethics committee of North-Kivu (No. 005/TEN/CENK/2020), and the study was carried out in accordance with the Helsinki declaration. Participants were anonymous, participation was voluntary, and there were no rewards for participating. All participants provided their tacit written consent or assent. We also obtained consent from parents or guardians of the minors included in this study. The manuscript was prepared in accordance with the Strengthening the Reporting of Observational Studies in Epidemiology (STROBE) checklist for cross-sectional studies [26].

## Results

### Sociodemographic and clinical characteristics of study participants

Most participants were aged between 18 and 50 (72.3%), men (59.4%), single (63.5%), Christian (71.8%), with moderate religious involvement (55.4%). Among patients presenting to psychiatric hospitals, 359/402 (89.2%) reported self-referral, and 204/400 (51%) attended the psychiatric hospital for follow-up or medication refills. The mean age of the first episode for psychiatric symptoms was 27.54 ± 12.43. Furthermore, 32% of participants attributed the cause of their mental health disorders to witchcraft, 30% to supernatural power, 12.5% to divine punishment. The first acute episode was reported at 30.5%; indicating the proportion of the sample who experienced a non-first episode of 69.6%. All characteristics of study participants are presented in Table 1.

**Table 1:** Baseline characteristics of study participants.

Characteristic	Statistic	
	**Frequency**	**Percentage**
**Age**	Mean: 31.02, SD: 5.14, Min: 16, Max: 65	
< 18	47	11.6
18-50	292	72.3
51- 65	65	16.1
**Sex**		
Male	240	59.4
Female	164	40.6
**Marital status**		
Married/cohabiting	106	26.2
Single	258	63.9
Divorced	27	6.7
Widower	13	3.2
**Occupation**		
Public	35	8.6
Private	220	54.5
Unemployed	149	36.9
**Educational level**		
University	26	6.4
Secondary	172	42.6
Primary	172	42.6
No formal education	34	8.4
**Religious orientation**		
Non-Pentecostal	318	78.8
Pentecostal	37	9.2
Other	41	10.2
None	8	2
**Religious attitude (N = 399)**		
Very religious	122	30.3
moderate	221	54.7
indifferent	56	14
**Mode of admission (N = 399)**		
Self-referred	365	89.2
Referred	34	10.8
**Age of first episode of psychiatric symptoms (years) (N = 404)**		
≤ 15	33	8
16–25	127	31.4
26–40	84	20.8
≥ 41	45	11.1
Unknown	115	28.5
**Evolutionary profile (N = 404)**		
First acute episode	123	30.4
Recurrent acute episodes	138	34.2
Progressive disease	117	29
Mixed	26	6.4
**Family history of mental disorders (N = 404)**		
None	189	46.8
Episodic disorder	156	38.6
Chronic disorder	33	8.2
Other	26	6.4
**Causal attribution (N = 404)**		
Natural	121	29.4
Divine punishment	50	12.9
Witchcraft	132	32.7
Other	16	4
unknown	85	21
**Attending the hospital for follow-up or medication refills (N = 400)**		
Yes	204	51
No	196	49

*N: number of participants and the denominator for percentages

### Determinants of first, second, third and fourth point for mental health seeking care

**Fig 1:**
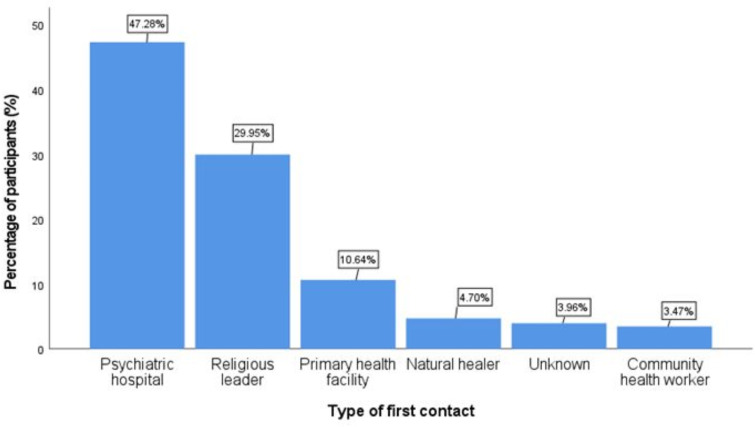
Distribution of participants by type of first contact for mental health care (n = 404).

**Fig 2:**
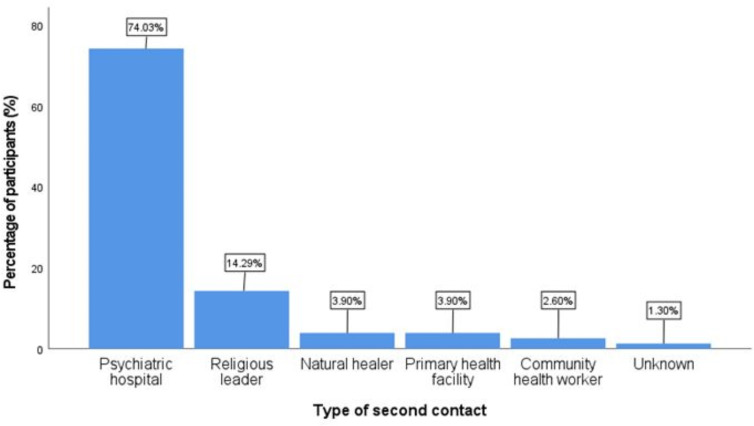
Distribution of participants by type of second contact for mental health care (n = 308).

**Fig 3:**
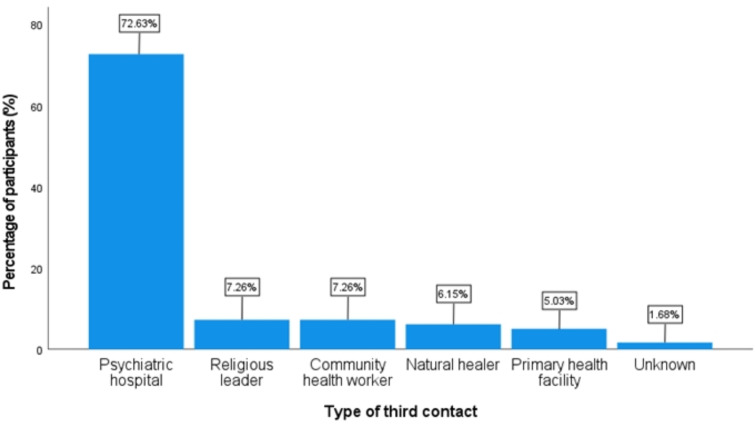
Distribution of participants by type of third contact for mental health care (n = 179).

**Fig 4:**
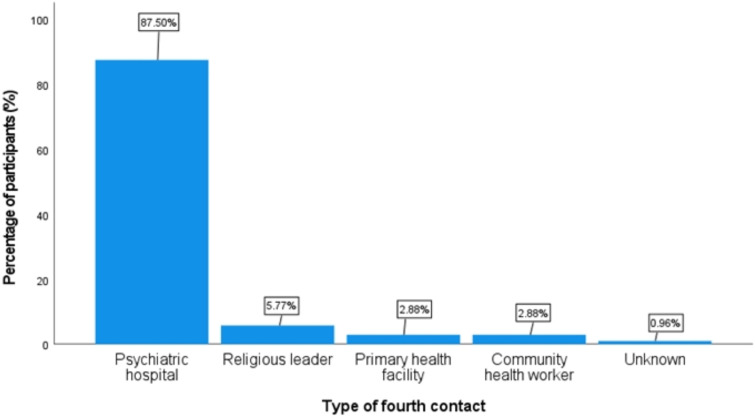
Distribution of participants by type of fourth contact for mental health care (n = 104).

Of participants whose first contact was a hospital (n = 404), nearly 1/3 (n = 121) reported subsequently consulting a religious leader. Psychiatric hospitals remained the most used facility for all contacts, followed by religious leaders. The self-reported sequences were subject, of not, to recall errors and, thus, do not establish knowledge of treatment effectiveness. In addition, 74% and 87.5% of participants attended psychiatric hospitals as their second and fourth care seeking options, respectively (see Figs 1–4).

### Stage specific predictors of seeking mental health services in non-primary health care facilities

Multivariate analyses revealed how attributing challenges to divine punishment was associated with higher odds of non-PHC first contact (aOR = 4.68, 95% CI [1.24–30.71] p = 0.040). A similar association was observed for witchcraft attributions (aOR = 2.35, 95% CI [1.06–5.45]. p = 0.040) and self-referral (aOR=6.37, 95%CI [2.82-14.59], p < 0.001). Self-referral was also associated with increased odds of seeking care in non-PHC settings on the second (aor = 3.25, 95%CI [1.08-8.85], p = 0.026) and third points of contact (aOR=9.57, 95% CI [2.00-51.49], p = 0.005). Patients with multiple episodes of psychiatric symptoms (aOR = 9.86, 95% CI [1.35-86.82], p = 0.028) had a higher likelihood of seeking mental healthcare in the non-PHC system at the third point of contact. Given the wide CI and multiple comparisons, this estimate is imprecise and should be interpreted cautiously. Having religious affiliation other than Pentecostal and non-Pentecostal (aOR=0.17, 95% CI [0.03-0.97], p = 0.026) and having a family history of mental disorders (aOR=0.06, 95%CI [0.01-0.39], p = 0.004) were associated with a decreased likelihood of seeking mental health care in non-PHC system. (see Table 2).

**Table 2:** Stage-specific predictors of seeking care in non-PHC versus PHC setting across the first, second, third and fourth or subsequent contacts.

	First contact for mental health						Second contact for mental health						third contact for mental health						fourth contact for mental health					
	PHC		NPHC				PHC		NPHC				PHC		NPHC				PHC		NPHC			
	n	%	n	%	aOR 95% CI [x–y]	P	n	%	n	%	aOR 95% CI [x–y]	P	n	%	n	%	aOR 95% CI [x–y]	p	n	%	n	%	aOR 95% CI [x–y]	p
Religion																								
Non-Pentecostal	40	12.6	278	87.4	Ref		15	6.2	226	93.8	Ref	–	14	10	126	90	Ref		5	6.7	70	93.3	Ref	
Pentecostal	8	21.6	29	78.4	0.52 [0.09-3.04]	0.5	1	3.1	31	96.9	1.99 [0.75-5.27]	0.17	3	15	17	85	2.24 [0.32-20.68]	0.48	0	0	13	100	0.98 [0.46-2.08]	0.96
Other	8	19.5	33	80.5	2.01 [0.87-4.65]	0.1	4	13.8	25	86.2	2.57 [0.95-7.00]	0.06	5	31.3	11	68.6	0.17 [0.03-0.97]	0.04	1	7.1	13	92.9	0.24 [0.15-0.74]	0.11
None	1	12.5	7	87.5	1.46 [0.23-9.29]	0.7	0	0	6	100	4.83 [0.99-11.18]	0.1	0	0	3	100	0.63 [0.29-1.36]	0.24	0	0	2	100	0.42 [0.20-0.88]	0.22
Religious attitude																								
Very religious	24	19.8	98	80.3	Ref	–	5	4.6	104	95.4	Ref	–	7	11.7	53	88.3	Ref		3	8.1	34	91.9	Ref	
moderate	25	11.3	196	88.7	1.58 [0.79 - 3.14]	0.2	12	7.7	143	92.3	67.47 [18.55-245.37]	0.06	12	13.6	76	86.4	NS		3	6.1	46	93.9	3.61 [1.08-12.07]	0.04
indifferent	8	14.3	48	85.7	0.91 [0.34 - 2.57]	0.8	3	7.5	37	92.5	0.18 [0.03-1.21]	0.08	3	10.3	26	89.7	NS		0	0	17	100	1.14 [0.36-3.58]	0.83
Address																	–							
Health zone	32	11.9	237	88.1	Ref		12	6	188	94	Ref	–	14	11.7	106	88.3	Ref		3	4.4	65	95.6	Ref	
Other health zone	24	18.5	106	81.5	0.78 [0.40 - 1.52)	0.4	8	7.7	96	92.3	0.85 [0.44-1.66]	0.64	8	14	49	86			3	8.8	31	91.2	2.54 [0.67-9.58]	0.17
Admission mode																								
referred	18	41.9	318	58.1	Ref		6	15.8	254	84.2	Ref	–	14	38.9	145	61.1	Ref		2	15.4	11	84.6	Ref	
self-referred	38	10.7	25	89.3	6.37 [2.82 -14.59]	<0.001	14	5.2	32	94.8	3.25 [1.08 - 8.85]	0.03	7	8.8	11	91.2	9.57 [2.00-51.49]	0.01	4	4.4	86	95.6	0.60 (0.96-3.70]	0.58
Development of the disease																								
First acute episode	21	17.1	102	82.9	Ref		5	7.6	61	92.4	Ref	–	5	19.2	21	80.8	Ref		2	12.5	14	87.5	Ref	
recurrent acute episodes	21	15.2	117	84.8	0.48 [0.19-1.18]	0.1	7	5.2	128	94.8	0.38 [0.11-1.31]	0.13	4	4.3	89	95.7	9.86 [1.35-86.82]	0.03	1	2	49	98	0.89 [0.16-4.99]	0.89
progressive disease	13	11.1	104	88.9	1.32 [0.53-3.30]	0.6	8	9.1	80	90.9	0.75 [0.20-2.86]	0.67	12	23.5	39	76.5	0.86 [0.16-4.44]	0.91	3	9.4	29	90.6	6.35 [2.07-19.53]	0.6
Mixed	2	7.7	24	92.3	1.40 [0.62-3.12]	0.4	0	0	19	100	0.49 [0.09-2.72]	0.42	1	11.1	8	88.9	2.20 [0.21-53.88]	0.54	0	0	6	100	4.77 [1.55-14.70]	0.07
Family history of mental health illness																								
None	28	14.8	161	85.2	Ref	–	6	4.4	132	95.7	Ref	–	8	11.6	61	88.4	Ref		3	7.7	36	92.3	Ref	
episodic disorder	23	14.7	133	85.3	0.24 [0.14-1.41]	0.5	11	8.9	113	91.1	0.47 [0.16 - 1.28]	0.21	6	7.5	74	92.5	1.37 [0.27-7.66]	0.71	3	6.3	45	93.8	2.45 [0.43-14.22]	0.32
chronic course	5	15.2	28	84.9	0.74 [0.42-1.30]	0.3	3	11.1	24	88.9	0.36 [0.09 - 1.83]	0.08	7	38.9	11	61.1	0.06 [0.01-0.39]	0.004	0	0	13	100	9.99 [1.24-65.13]	0.13
Other	1	3.9	25	96.2	0.77 [0.43-1.38]	0.4	0	0	19	100	NS		1	8.3	11	91.7	1.12 [0.12-28.47]	0.93	0	0	4	100	1.47 [0.04-6054]	0.8
Causal attribution																								
Natural	23	19	98	80.5	Ref		4	4.7	82	95.4	Ref	–	3	6.7	42	93.3	Ref		1	3.9	25	96.2	Ref	
Divine punishment	3	6	47	94	4.68 [1.24-30.71]	0.04	1	2.9	33	97.1	6.79 [3.00-15.40]	0.69	1	4.6	21	95.5	4.3 [0.33-128.67]	0.33	0	0	10	100	4.13 [0.50-34.02]	0.19
Sorcery	16	12.1	116	87.9	2.35 [1.06-5.45]	0.04	10	9.2	99	90.8	1.67 [0.78-3.57]	0.63	8	12.1	58	87.9	1.32 [0.20-8.29]	0.84	3	7.7	36	92.3	5.88 [0.89-38.91]	0.07
other	2	12.5	14	87.5	1.48 [0.35-10.22]	0.1	0	0	11	100	NS	–	1	16.7	5	83.3	0.89 [0.05-32.65]	0.9	0	0	3	100	6.16 [0.19-17.64]	0.13
unknown	13	15.3	72	84.7	1.21 [0.53-2.83]	0.7	5	7.4	63	92.7	3.85 [1.80-8.22]	0.3	9	22.5	31	77.5	0.26 [0.04-1.35]	0.12	2	7.7	24	92.3	0.56 [0.01-23.93]	0.76

aOR: adjusted odd ratio; PHC: primary health care; NPHC: non-primary health care; p: p value; CI: confidence interval; Outcome coded as 1 = non-PHC and 0 = PHC at each contact stage; aOR>1 indicates higher odds of seeking care in non-PHC settings; aOR<1 indicates decreased likelihood of seeking mental health care in non-PHC settings.

## Discussion

In the current study, we sought to describe the patterns and factors associated with pathways to mental healthcare, with an emphasis on how religious beliefs and referral patterns are associated with the use of PHC in war-torn Eastern DRC where mental health services are not fully integrated in the PHC system. We found that most participants presenting to psychiatric hospitals were self-referred, which aligns with findings from previous research in similar (i.e. conflict zone) settings [27,28]. Determinants of self-referral to psychiatric hospitals included the accessibility of the hospitals, the availability of trained mental health specialists, and a well-established referral system linking the PHC and psychiatric hospitals [29,30].

Access to psychiatric hospitals in low-income countries is impaired by socio-economic status, cultural norms, and religious beliefs. Specifically, we found that one in four or 25 percent of participants presenting at PHC facilities have a mental health challenge [31]. In our cohort, the results indicate that eight to ten people with mental health disorders attended psychiatric hospitals after seeking help from traditional healers and religious leaders, echoes the findings of studies conducted by Nakku and colleagues [32] and Eagle et al [33]. Moreover, the high rate of non-first episode of 69.6% in our sample could result of patients abandoning medical treatment in favor of religious centers during periods of remission. In Eastern DRC, religious beliefs and cultural norms influence health-seeking behaviors, as highlighted by our previous study showing that nearly six in ten people seeking spiritual help in religious centers had psychiatric symptoms [5], regardless of them ever being admitted or not. This is, further, supported by the proportion of patients admitted to psychiatric hospitals who returned to religious centers for their second contact. In DRC, like in several Sub-Sahara African countries, patients with mental health challenges usually spend months or years navigating religious or traditional pathways or PHC facilities. These individuals only reach psychiatric hospitals once their condition has become chronic and severe, with impaired quality of life and complications such as suicide attempts. Their behavior is better explained by high proportion of non- Pentecostal participants in our sample, which aligns with Eagle et all’s work that emphasized how both Pentecostal/Charismatic and non-Charismatic Protestant pastors in Eastern DRC endorsed a combined approach to treating symptoms of depression, which supports the prevalence of both spiritual and medical interventions [33].

Several factors influence citizens’ decisions to seek treatment for mental health disorders, including severity of illness, suitability of treatment, and sociocultural practice and religious denomination. Approximately one-third of participants indicated they had initially consulted a religious leader as their first point of contact, after which a notable proportion (14.29%) continued to seek additional support from a religious leader (i.e., second contact), with fewer presenting to religious leader at third and fourth. This could explain the high proportion of the sample experiencing a non-first episode in this cohort because of patients abandoning medical treatment in favor of religious leaders during periods of remission. The finding suggests religious leaders can serve as effective partners in facilitating access to formal mental health care, given they may practice traditional medicines and, most importantly, provide an alternative to psychiatric treatment in countries with few mental health specialists [5,12]. Moreover, our findings reveal, after first contact in seeking mental health care in psychiatric hospitals, a notable proportion of participants sought additional support from religious leaders and traditional healers at the second contact, with fewer doing so at the third and fourth contacts. Religious leaders and traditional healers are of paramount roles in the management of mental health challenges even when psychiatric treatment is used by individuals with mental health challenges in psychiatric hospitals [34].

Understanding interpretations of mental health needs influences help seeking behaviors. Thus, for example, scholarship has found attributing mental health disorders to divine punishment and witchcraft encourages trust in the therapeutic effect of spirituality and religion, and the rejection of pharmaceutical interventions for mental health disorders [35]. Aligning with existing literature [30,36,37], we too found believing that compromised mental health results from supernatural forces, such as divine punishment or witchcraft, appears associated with reliance on PHC systems, especially in the first point of care, in addition to self-referrals. Stigma, religious beliefs and practices, and a lack of accurate information about mental health challenges influence how people interpret mental health problems and if they seek care from a religious leader [38], thus shaping how individuals perceive and seek help for mental health in low-income and low-resource settings [39]. Existing evidence emphasizes how having unskilled medical professionals in PHC and psychiatric hospitals exacerbate the influence of cultural determinants of health for those in dire need of intervention, thus constituting a major contributing factor for the poor utilization of mental health services, particularly in low-income countries [39,40]. Addressing stigmas using culturally appropriate mental health education and establishing a living interaction between medical professionals, traditional healers and religious leaders may, in consequence, promote earlier contact with appropriate mental healthcare services. Due to their role in care pathways, policymakers should consider establishing evidence-informed interventions that target the provision of biopsychosocial therapies associated with spiritual care for people seeking help in primary health care settings [41].

### Late entry to psychiatric care and need of collaborative model between religious leaders and medical professionals

Our findings show how the current medical system in the DRC, the PHC system, is too often bypassed because the model does not fully account for the cultural determinants of health, especially for those with metal health needs. A significant proportion of individuals seeking religion informed interventions prior to and after seeking care in psychiatric hospitals reveals a need for collaborative care provision from religious and medical leaders. Thus, a holistic model of care that considers religious and cultural determinants of health, combining spiritual, traditional, and biomedical treatment approaches, is necessary for the betterment of and appropriate management of mental health challenges among individuals in conflict zones of DRC. Such a collaboration constitutes an evidence-informed strategy which could be both accessible and feasible, thus promising, for DRC. The Emerald consortium, involving six countries (Ethiopia, India, Nepal, Nigeria, South Africa, and Uganda) demonstrates how strengthening policy, legislation, and health system capacity can, if the cultural importance of religious leaders and traditional healers is considered, enable the integration of mental health into primary care [42]. In Northern Ghana too, efforts to integrate traditional healers and biomedical providers show how trust-building, clear recognition of healers, and structured communication mechanisms help overcome barriers to collaboration [43]. Additionally, global evidence from recent collaborative models indicates that involving traditional healers in training and formal referral networks improves mental health outcomes and increases link to psychiatric services [44]. Drawing on these examples, establishing standardized communication channels between medical professionals and religious leaders, while strengthening the capacity of PHC workers, is a realistic and context-appropriate step toward reducing reliance on religious and traditional healing centers among people with mental health needs. Finally, good mental health is a universal right, and additional efforts are needed to support individuals suffering from mental health disorders, particularly those living with the added burdens of war and civil unrest.

### Study limitations

The sample consists only of psychiatric hospital patients, excluding individuals within communities outside of such, which reduces the voices of those who did not receive care from psychiatric facilities. Additionally, the study being cross-sectional study, rather than longitudinal, does not enable the establishment of a cause-effect relationship. Generalizability too is hindered by the non-randomized sampling method and the lack of controls in the analysis. In response, longitudinal studies and qualitative work nuancing context are warranted, particularly if inclusive of people living in communities, primary health care providers, and individuals’ experiences with and interpretations of psychiatric hospitals. This is necessary given our participants were exclusively patients attending psychiatric hospitals regardless of the number of admissions or episodes. Further limitations include how participants pathway-to-care histories were based solely on self-report, susceptible to recall and social desirability biases, as well as potential misclassification regarding service provision attempts and processes, particularly when help-seeking trajectories are intricate and involve numerous care contacts. Our study relied on pretested self-reported questionnaires because of a lack of validated scales detailing religious involvement. Further studies should aim to validate these tools for the individuals living in conflict zones such as the Eastern DRC. The number of predictors included in the multivariate models may have been large relative to the number of outcome events at some contacts stage, such as attributing challenges to divine punishment was associated with higher odds of non-PHC first contact with broad confidence ranges. This may have resulted in overfitting, unstable parameter estimates, and wide confidence intervals, thereby reducing the precision of some adjusted odds ratios. In particular, the broad confidence intervals observed for some predictors suggest limited statistical power and possible sparse-data bias. Therefore, associations identified in later-stage models should be interpreted cautiously. In addition, standard model diagnostics such as goodness-of-fit statistics, pseudo-R², and formal checks for multicollinearity between predictors were not conducted, which may further limit the robustness of our findings. Because separate logistic regression models were fitted for each contact stage, the analysis did not explicitly account for within-person correlations across repeated contacts over the care pathway. Therefore, the results should be interpreted as snapshots at each stage rather than as longitudinal patterns. Despite this, the method offers valuable insights into how care-seeking behaviors evolve across different points in the care pathway. Future studies with larger sample sizes and more rigorous model diagnostics are warranted to confirm these associations, perhaps a mixed-effects model could be used in future analyses. Sociodemographic variables such as age, sex, marital status, and employment status were examined but showed no significant association with the four points of contact in bivariate analysis, and education was excluded during stepwise logistic regression, which warrants additional research intended to reveal why. Nevertheless, the absence of these socioeconomic indicators in the final models may still limit interpretation, as they are known from other settings to influence pathways to care. Furthermore, the width of the confidence intervals does require caution as it suggests a sufficient sample size is lacking, which could affect the estimated correlations analyzed through the regression models.

## Conclusion

Our findings reveal a significant underutilization of PHC for mental health needs in conflict zones, attributable, at least in part, to a failing adequately accounting for the cultural determinants of mental health. Instead, patients favored self-referral to psychiatric hospitals or religious leaders due to their strong cultural and religious beliefs (i.e., attributing mental health disorders to witchcraft, supernatural powers, or divine punishment), as well as a lack of integrated mental health services within the PHC system in conflict zones of DRC. While the DRC’s situation is extreme due to war and civil unrest, there remains an undeniable need for a collaborative model between religious leaders and medical professionals intended to improve care pathways in these conflict zones. Thus, much can be learned from drawing on models that have been successfully used with positive outcomes elsewhere. Finally, building the capacity of frontline workers, improving referral links, and promoting culturally sensitive education could help make mental health care more accessible and responsive in fragile settings.

## Supporting information

S1 Table Distribution of study participants per psychiatric hospital. (DOCX)

S1 Data Dataset. (XLSX)
