# Supplementary material for: Beliefs, referrals, and mental healthcare pathways in the Eastern Democratic Republic of Congo
Source: PLOS Glob Public Health. 2026 Jul 2;6(7):e0006715. doi: 10.1371/journal.pgph.0006715 (PMC13327247; doi:10.1371/journal.pgph.0006715)
Supplement: S1 Table — (DOCX) [file pgph.0006715.s001.docx]

S1 Table: Distribution of study participants per psychiatric hospital

| Mental hospital | Frequency | Percentage |
| --- | --- | --- |
| Polycliniques Sainte Croix | 84 | 20.8 |
| Cap Salama | 73 | 18.1 |
| Cepima | 58 | 14.4 |
| Centre Muyisa | 54 | 13.4 |
| Centre la guérison | 43 | 10.6 |
| Ceresame | 34 | 8.4 |
| Cediar | 28 | 6.9 |
| Bora uzima | 20 | 5.0 |
| Notre dame de lourde | 10 | 2.5 |
