# Supplementary material for: Beliefs, referrals, and mental healthcare pathways in the Eastern Democratic Republic of Congo
Source: PLOS Glob Public Health. 2026 Jul 2;6(7):e0006715. doi: 10.1371/journal.pgph.0006715 (PMC13327247; doi:10.1371/journal.pgph.0006715)
Supplement: Attachment — Submitted filename: responses to reviewers 23.10.2025.pdf [file pgph.0006715.s003.pdf]

Manuscript ID: PGPH-D-25-01441

Manuscript title: Entry Points to Mental Healthcare: The Influence of Beliefs and Referrals on the Use of the Primary Healthcare System in North Kivu Province, Eastern Democratic Republic of Congo

RE: Corrections in response to reviewers' comments

Dear Editor

Greetings

Thank you for considering our manuscript and for the comments to make it perfect.

Please find in the table below the responses to reviewer's questions and we hope that the current version meets the expectations of the reviewer and editorial teams.

| Number | Questions from reviewer | Answer from author | Page |
|---|---|---|---|
| Reviewer #1 | | | |
| A.Title | | | |
| 1 | Consider shortening the title for clarity, e.g., 'Beliefs, Referrals, and Mental Healthcare Pathways in Eastern DRC' to make it more concise while retaining the main elements. | We have revised the title for a short and concise title | Page 1 |
| B. Abstract | | | |
| 2 | Issue: The abstract is lengthy with some redundancy (e.g., repeating "psychiatric hospital" multiple times).<br>Suggestion: remove redundancy, streamline background; focus on key statistics, main predictors, and implications. Include important statistical results (aORs, p-values) for stronger impact. Ensure all abbreviations (e.g., PHC) are defined on first use. | We have updated the abstract removing some redundancy, focusing on key statistics, including important statistics and defining abbreviations. | Page 2 |
| C. Introduction (lines 52–57) | | | |
| 3 | Correct grammar in paragraph 1 ("play" → "plays"), and ensure consistent tense usage. | We have removed the sentence after major revision in the paragraph | Page 3 |
| 4 | Shorten long sentences in paragraph 3 for better readability. | We have reviewed the paragraph 3 for better readability | Page 3 |

| | | | |
|---|---|---|---|
| 5 | End the introduction with a one-sentence, clearly stated research objective/hypothesis. | We reviewed the end of the introduction as needed | Page 4 |
| 6 | lines 66–77<br>Issue: Long sentences with multiple clauses reduce clarity.<br>Suggestion: Break into shorter sentences for readability. | We have reviewed the sentence for better readability | Page 3 |
| 7 | Lines 96–102<br>Issue: Study aim is stated but not as a clear, concise research objective.<br>Suggestion: End the Introduction with a specific, one-sentence statement of objectives or hypothesis. | We reviewed the end of the introduction stating the aim of the study in one-sentence as needed | Page 3 |
| D. Method | | | |
| 6 | Rewrite hospital inclusion description for clarity (paragraph 2). | We have revised hospital inclusion in the paragraph as needed | Page 3 |
| 7 | Simplify questionnaire description (paragraph 4). | We have revised the paragraph 4 as needed | Page 4 |
| 8 | Justify or cite categorization of religiosity (paragraph 6). | The 3 categories of religiosity have been justified by citing related references | Page 4-5 |
| 9 | Specify all variables used in regression and cite literature for selection (paragraph 9). | We have specified the free categories of variables used in regression and cited related literature | Page 5 |
| 8 | Lines 105–118; Study Setting<br>Issue: Minor grammar and clarity issues (“resulting in Polycliniques…” is awkward).<br>Suggestion: Rewrite for clarity, e.g., ‘The selected hospitals included… These facilities met inclusion criteria of…’. | We have revised the paragraph as needed | Page 3 |
| 9 | lines 131–143; Data Collection<br>Issue: Phrase ‘never been published’ in describing questionnaire is redundant.<br>Suggestion: Simply state ‘a structured questionnaire developed for this study.’ | We removed the phrase “never been published” and replace it by “We developed the study survey” | Page 4 |

| | | | |
|---|---|---|---|
| 10 | lines 153–158; Religiosity Measures<br>Issue: Definition of 'very religious' as 'more than five days a week' may need referencing or justification.<br>Suggestion: Cite supporting literature for these cut-offs or note they were based on pilot testing. | The 3 categories of religiosity have been justified by citing related references | Page 4 |
| 11 | lines 172–182; Statistical Analyses<br>Issue: Variables included in regression are said to be 'chosen based on existing studies' without listing them fully.<br>Suggestion: Specify variables and cite relevant studies for transparency. | We have specified the free categories of variables used in regression and cited related literature | Page 5 |
| E. Results | | | |
| 12 | lines 194–199; Clinical Characteristics<br>Issue: Reported relapse rate (69.5%) is important but not discussed in results narrative.<br>Suggestion: Briefly contextualize its significance in the results section. | We have contextualized by indicating that "The first acute episode was reported in 30.45; indicating the relapse rate of 69.45%" in Table 5 | Page 7 |
| 13 | Tables 2–4<br>Issue: Inconsistent p-value and CI formatting across tables/figures.<br>Suggestion: Standardize format per PLOS style (e.g., p=0.05, 95% CI [x–y]) in all tables. | We standardized p value and 95% CI as required by the PLOS style. | Page 9-11 |
| F. Discussion | | | |
| 14 | lines 229–237<br>Issue: Repeats content from abstract nearly verbatim.<br>Suggestion: Focus on interpreting findings rather than restating them. | The discussion section has been revised to avoid repetition of the abstract and instead provide a deeper interpretation of the findings, emphasizing their implications within the broader literature and contextual realities. | Page 12-13 |
| 15 | lines 268–277); Integration Recommendation<br>Issue: Long sentence chains make the recommendation difficult to | We have revised the paragraph as recommended resulting in the free separated idea mentioned. | Page 12-13 |

| | | | |
|---|---|---|---|
| | follow.<br>Suggestion: Split into separate sentences: one for holistic model advocacy, one for integration, one for collaboration mechanisms. | | |
| 16 | 16. Lines 278–291; Policy Implications<br>Issue: Important points on stigma and cultural beliefs, but needs references to back claims (e.g., ‘health workers… do not have adequate training’).<br>Suggestion: Add citations or state that this is based on study findings. | We have provided references that support the idea. | Page 12 |
| G. Limitations | | | |
| 17 | Lines 292–305<br>Issue: ‘Quantitative approached’ correct ‘approach’ to ‘approach’, clarify whether 404 is truly low sample size for cross-sectional design<br>Suggestion: Revise wording to reflect actual limitation and avoid misleading reader. | We have revised the limitation section as needed by the reviewer. We have also indicated that the convenient sample is the limit, not the sample size, which is sufficient. | Page 13 |
| H. Conclusion | | | |
| 18 | Lines 306–312<br>Issue: Restates results without linking back to broader literature or practical implications in depth.<br>Suggestion: End with a strong recommendation for policy/practice based on findings, i.e. Strengthen conclusion with specific, actionable recommendations. | The conclusion was revised to emphasize practical and policy implications, highlighting integration of mental health into primary care, intersectoral collaboration, and community education in conflict settings. | Page 13 |
| I. Other general Points. | | | |
| 19 | Language: Throughout, minor grammar and syntax issues need correction (subject–verb agreement, tense consistency). Recommend professional English editing. | The entire manuscript has been carefully reviewed and edited for grammar, syntax, and tense consistency | Whole manuscript |

| | | | |
|---|---|---|---|
| 20 | Reporting Guidelines: Add explicit statement in Methods noting adherence to STROBE checklist for cross-sectional studies. | We have stated the adherence to STROBE checklist for cross-sectional studies in method section | Page 5 |
| 21 | Data Availability: PLOS encourages depositing anonymized data in a public repository rather than 'available on request.' | We have indicated that the dataset has been deposed in the manuscript and we have uploaded it as a supporting information 3 | Page 174 |
| 22 | Reference List: Check for over-reliance on the authors' own work and ensure balanced citation of independent studies. | We have revised the reference list | 14-17 |
| Reviewer #2 | | | |
| Major Comments | | | |
| 1. Writing Clarity and Structure | The manuscript contains multiple grammatical errors, awkward phrasing, and inconsistent terminology (e.g., "war-tone" vs "war-torn"; "non-conventional hospitals" vs "non-primary healthcare hospitals" vs "psychiatric hospitals"). | We have systematically replaced "War-tone" by the appropriate term "war-torn", as well as non-conventional hospitals by non-primary health care hospitals. Psychiatric hospitals still used as a specific entry point in the pathway. | Whole manuscript |
| | Many sentences are overly long and repetitive, reducing clarity for an international audience. | We have revised the whole manuscript as needed by the reviewer | Whole manuscript |
| | The Abstract and Discussion in particular repeat the same statistics without adding new interpretation. | We have revised as needed by the reviewer | Page 2, 12-13 |
| | Clearer definitions are needed for "non-conventional hospital," "primary healthcare hospital," and "psychiatric hospital" early in the Methods to avoid confusion. | Operational definitions of the concept have been mentioned in introduction and method sections. | Page 3-5 |
| 2. Contradictions in Findings | Self-referral was reported as 89.2% (Results) and also described as 47.3% first contact with psychiatric hospitals (Figure 1), which appears inconsistent. Authors should reconcile or explain this discrepancy. | We have revised the whole manuscript as needed by the reviewer by removing the discrepancy.<br>First, these are two different variables. The first relates to the mode of admission to the place where the patient was encountered, i.e., the | Page 7) |

| | | | |
|---|---|---|---|
| | | psychiatric center, with two possibilities (either referred to by other healthcare providers or not). The second variable relates to the first point of contact, i.e., the first time the patient sought care for the mental disorder from which they suffer. | |
| | In the Discussion, “one in three” patients seeking religious care after hospital treatment is highlighted as a major finding, but this pattern is not clearly supported by the presented data tables. | We have revised the manuscript as needed by the reviewer.<br>The sentence has been corrected. In fact, it refers to the proportion of people who sought help from servants of God at first contact (Figure 1). This result has been better written and discussed. | Page 2, 8 |
| | The proportion attributing illness to “God’s punishment” (12.85%) is comparatively small, yet it is discussed as a dominant explanatory factor without proportional emphasis on witchcraft (32.67%) or natural punishment (29.95%). | We have revised the manuscript as needed by the reviewer As the two categories. | Page 12 |
| 3. Methodological Rigor | Sampling bias – The sample consists only of psychiatric hospital patients, excluding individuals who never access these facilities. This likely overestimates the proportion of non-primary healthcare pathways. | We have revised the manuscript as needed by the reviewer by adding it details in the limitation section | Page 13 |
| | Instrument validation – The survey tool was self-developed; while piloted, no psychometric properties (e.g., reliability coefficients, validity evidence) are provided. | Although there is no validated study, we have noted that the WHO encounter form has been used in several studies in the African context (like Ethiopia), the pathway has been characterized as both possible and acceptable for gathering pertinent pathway | Page 13 |

| | | | |
|---|---|---|---|
| | | data. The issue has been discussed in the limitation section. | |
| | Recall bias – Pathway-to-care histories rely entirely on self-report, which is particularly vulnerable to recall error in multi-contact health-seeking patterns. This is not discussed as a limitation. | This has been acknowledged in the limitation | Page 13 |
| | Variable selection for regression – Factors "chosen based on existing studies" is too vague. Authors should specify model-building strategy and justify inclusion/exclusion. | We have specified the free categories of variables used in regression and cited related literature. | Page 5 |
| 4. Results Reporting | Inconsistent sample sizes: Table 2 reports n=411, but Methods specifies n=404. | We have corrected the inconsistency of sample size in table 2. | Page 7 |
| | Regression results have extremely wide confidence intervals (e.g., aOR=9.86, CI=1.35–86.82), indicating unstable estimates likely due to small subgroup counts—yet no cautionary interpretation is provided.<br><br>Missing model diagnostics (goodness-of-fit, pseudo-$R^2$) and no check for multicollinearity between predictors. | We have discussed the issues of wide confidence intervals in regression results as a limitation of the study and of the standard model diagnostics. | Page 9 |
| | Figure 1 lacks axis labels, clear legends, and an explanation of what "n" values represent at each stage. | We have revised Figure 1 as needed by the reviewer | Page 8 |
| 5. Interpretation and Overgeneralization | The authors make causal-sounding claims (e.g., "beliefs increase the odds") from cross-sectional association data without sufficient caution. | We have mentioned caution in the interpretation of regression results as associated factors with higher or low odds of seeking mental health care in the non-primary health care system. | Page 12-13 |
| | Recommendations to integrate religious leaders into formal care systems are not directly supported | Due to the study design, we have cautionary stated that the findings suggest that | Page 13 |

| | | | |
|---|---|---|---|
| | by intervention evidence from this study. | collaborative models between religious leaders and PHC and mental health workers could be an effective strategy for improving access to mental healthcare. | |
| | Conclusions generalize to "most patients with mental disorders in war-torn zones" despite only sampling those attending psychiatric hospitals. | We have revised the manuscript as needed by the reviewer | Page 13 |
| 6. Limitations | Important biases— social desirability bias, and misclassification—are missing from the limitations section. | We have discussed social desirability bias and misclassification in limitation section. | Page 13 |
| | No discussion on the absence of socioeconomic variables (income, education) in regression models, despite their likely influence. | We have mentioned the absence of socioeconomic variables in the regression model as a possible limit of the study due to the absence of association at bivariate step of the analysis. | Page 13 |
| | Missing data handling is not described. | We have described the exclusion of participants with missing data and the absence of data imputation. | Page 4 |
| Minor Comments | Abstract: Clarify the meaning of "non-conventional hospitals" and ensure percentages match those in Results. | We replaced "non-conventional hospital" by non-primary care system and clarified that it refers to mental health facilities outside the primary health care system and verified that all percentages now match the Results section. | Page 2 |
| | Introduction: The review of literature is relevant but would benefit from more DRC-specific policy context (e.g., mhGAP integration efforts). | We have revised the manuscript to include a discussion of DRC-specific mental health policy context, including ongoing challenges to integrate mhGAP into primary healthcare services. | Page 3, 4 |

| | | | |
|---|---|---|---|
| | Methods: Specify how missing values were addressed and whether any data imputation was performed. | We have described the exclusion of participants with missing data and the absence of data imputation. | Page 4 |
| | Tables: Include denominators for percentages and ensure all abbreviations (PHC, aOR) are defined in footnotes. | We have revised the manuscript to include denominators for all reported percentages and ensured that all abbreviations, including PHC and aOR, are clearly defined in footnotes. | Whole manuscript |
| | Discussion: Reduce repetition of numerical results already presented; focus on interpreting unexpected or contextually important findings. | We have revised the discussion as needed by the reviewer. | Page 14, 15 |
| | Language: Correct typographical errors (“necessarily modifications” → “necessary modifications”, “tone” → “torn”). | We have corrected the mentioned typographical errors. | Whole manuscript |
| | References: Ensure consistent formatting according to PLOS style (some references are missing full journal details) | We have revised as needed by the reviewer. | 14-17 |
| Reviewer #3 | | | |
| 1. | Introductory section may be further developed to add some more relevant literature. | We have added some more relevant literature focused on DRC context. | Page 3 |
| 2. | Implications of the study may galvanize other readers to follow same type of study | Thank you. We do hope so. | |
| Reviewer #4 | | | |
| Overall | Several sentences exceed 30 words; shortening would improve clarity. Consistent tense usage (past for methods/results, present for implications) should be ensured. | We reviewed the manuscript ensuring the long sentences are shortened, and consistent tense is used in different section. | Whole manuscript |
| Abstract | Well-structured, but some sentences are long and could be more concise. | We have shorted long sentences in the abstract for more consistency. | Page 2 |
| | L28–31: Combine to avoid redundancy in stating the knowledge gap. | We have combined sentences to be more concise. | Page 2 |

| | | | |
|---|---|---|---|
| | L34: “patients who attending” → “patients attending.” | We have corrected the grammar errors as mentioned. | Page 2 |
| | L46–47: “due to such determinant” → “due to determinants such as.” | We have revised the paragraph dropping the missing mentioned. | Page 2 |
| Introduction | L52: “Although primary health care play” → “plays.” | We have revised the paragraph, then the mentioned clause has been dropped. | Page 3 |
| | L63–65: Sentence structure awkward; revise for clarity (“have not centered on people in war-torn settings or on patterns…”). | We have revised the paragraph dropping the mentioned clause. | Page 3 |
| | L78–81: Strong justification with prevalence data. | We have included a strong justification of the prevalence data. | Page 3 |
| | L87–94: Clear explanation of theoretical framework; well-linked to study aim. | We have clearly explained the framework and linked it to the study aim. | Page 3 |
| | L97–101: Slight grammatical issues; tighten for clarity. For example, “aimed to the patterns” → “aimed to examine the patterns.” | We reviewed the manuscript and corrected all the grammatical errors we identified. | Page 3 |
| Methods | L106–113: Consider moving the rationale for hospital selection to a bulleted list for clarity. | We rewrote the paragraph as required. | Page 3 |
| | L138–141: “Never been published” not necessary unless relevant for novelty claim. | We dropped the clause in the revised paragraph. | Page 4 |
| | Translation and piloting described well; however, specify inter-rater reliability or quality control in data collection. | We added how inter-rater reliability and quality control in data collection was assured. | Page 4 |
| | Variables section is detailed; some long sentences could be split for readability. | We rewrote the paragraph shortening some long sentences for readability. | Page 5, 6 |
| | Statistical analysis description appropriate; mention if model diagnostics were performed | We noted the model diagnosis performed. | Page 5 |
| Results | Tables are informative; ensure consistent decimal formatting and labeling. | We considered two decimals in the whole tables in the manuscript. | Whole manuscript |
| | L195–199: Minor grammar issues (“relapse rate was 69.5%”). | We revised and edited. | Page 7 |

| | | | |
|---|---|---|---|
| | Figure 1 reference is good; ensure high-quality, clearly labeled figures for publication. | We clearly labeled the figures. | Page 8 |
| | L215–227: Well-presented predictors; however, Table 5 should be integrated in-text with more interpretation. | We integrated table 5 into the text and added more interpretation. | Page 11 - 13 |
| Discussion | L229–237: Nicely summarizes key findings upfront. | We have summarized key findings upfront. | Page 12 |
| | Well-linked to literature, but several sentences are long and contain multiple ideas (e.g., L239–246). Breaking these will improve flow. | We broke up sentences in the discussion section to improve flow. | Page 12-13 |
| | Strong point on post-hospital religious center visits; valuable policy implication. | Thank you. | |
| | L268–277: Good integration of recommendations for collaborative models. Could emphasize feasibility and examples from similar contexts. | We rewrote the paragraph emphasizing feasibility and using examples from similar contexts. | Page 13 |
| | L278–291: Highlights cultural determinants effectively; could add a brief comment on stigma reduction strategies. | We have added a brief comment on stigma reduction strategy in the paragraph. | Page 12-13 |
| | L292–305: Limitations are well-acknowledged; however, some phrasing is defensive ("may affect the responses of participants"). Instead, frame as neutral methodological constraint. | We rewrote the limitation section avoiding defensive formulations as required. | Page 13 |
| | Could mention potential recall bias due to self-reported pathways. | We have discussed recall bias in the limitation section. | Page 13 |
| Reviewer #5 | | | |
| | Firstly, the main claim of the study implies a causal association between beliefs and referrals and use of primary healthcare system and yet the study design is cross-sectional. This is Moreso given that the sampling method had no randomization and introduces potential bias. I would suggest that the authors consider a more | We appreciate the reviewer's observation. We have revised the manuscript to consistently describe the findings as associations rather than causal relationships, in line with the cross-sectional design. | Whole manuscript |

<table>
<tr><td></td><td>conservative claim and do so consistently from title, abstract to discussion.</td><td></td><td></td></tr>
<tr><td></td><td>L230 (“we sought to determine the patterns and determinants…”)<br><br>Issue: “Determine” implies causality.<br><br>Possible Fix: “we sought to describe patterns and identify factors associated with pathways…”</td><td>We have rewrote the sentence as required focusing on describing patterns and the associated factors.</td><td>Page 11</td></tr>
<tr><td></td><td>“After treatment successes” implies efficacy and temporal sequence not established.<br><br>Fix (replace L235–237):<br>“Among those presenting to psychiatric hospitals, 359/402 (89.2%) reported self-referral. Of those whose first contact was a hospital (n= [insert]), ~1/3 reported subsequently consulting a religious center. These self-reported sequences are subject to recall error and do not establish treatment effectiveness.”</td><td>We rewrote the paragraph replacing the clause mentioned by the reviewer.</td><td>Page 16 (line 260 – 265)</td></tr>
<tr><td></td><td>While the statistical approach is well suited to cross-sectional categorical data, the conclusions that are arrived at from the significance are faulty in that they establish causality.</td><td rowspan="2">We thank the reviewer for raising this and agree that participants were followed across multiple contact points. To address this, we conducted separate binary logistic regression models for each contact level, each model contains only one observation per participant, thus respecting the assumption of independence.<br><br>This approach allows us to identify factors specifically associated with choosing non-primary care services at</td><td></td></tr>
<tr><td></td><td>While multinomial logistic regression is appropriate for categorical outcomes, the analysis overlooks repeated measures, clustering by hospital, sparse data bias, and multiple comparisons. Without addressing these and clarifying definitions, results risk bias and overinterpretation. Re-analysis with robust, stage-aware methods and cautious interpretation is required.</td><td>Page 5</td></tr>
</table>

<table>
<tr>
<td></td>
<td>Take care of repeated measures, the same participant may contribute data to multiple contact stages, but the model treats these as independent. This can underestimate standard errors. A stage-aware method (e.g., GEE or multilevel modelling with participant ID and hospital as clusters) would be more robust.</td>
<td rowspan="3">each stage of the care pathway, which may differ between first and subsequent contacts.<br><br>While mixed-effects models could be considered to account for within-participant correlations across contacts, our approach ensures interpretability of stage-specific associations, which is central to our study objectives.<br><br>Further we mention precaution in interpretation in the limitations.</td>
<td rowspan="3"></td>
</tr>
<tr>
<td></td>
<td>Possibly consider clustering by hospital, participants are nested within nine hospitals, which may share referral patterns or patient profiles. Ignoring this clustering risks overstating precision. Cluster-robust SEs or random effects are recommended.</td>
</tr>
<tr>
<td></td>
<td>L216–220<br><br>Unit of analysis and model not fully clear for stage-specific outcomes; repeated measures likely (same person contributes multiple “contact points”), yet independence is implied.<br><br>This is an issue because it violated independence inflates Type I error and narrows CIs.<br><br>You may correct by stating the modeling approach and address dependence (GEE or mixed-effects) and hospital clustering; clarify that stage models are conditional on reaching that stage.<br><br>My suggested way to correct is to add after L216<br>“We modeled stage-specific outcomes conditional on participants having reached that stage. To account for repeated measures across stages and</td>
</tr>
</table>

| | | | |
|---|---|---|---|
| | clustering by hospital, we used [GEE with logit link and exchangeable correlation / mixed-effects logistic regression with random intercepts for participant and hospital]. Results are reported as adjusted odds ratios (aOR) with cluster-robust 95% CIs.” | | |
| | L217–218 (“God’s punishment” aOR=4.68, CI 1.24–30.71, p=0.05)<br><br>Issue: Very wide CI and borderline p; precision is low; multiple testing not acknowledged.<br><br>Why: Risk of false positives and sparse-data bias.<br><br>Action: Flag imprecision; consider penalized methods (Firth) or category collapse if sparse.<br><br>You may replace sentence: “Attributing challenges to God’s punishment was associated with higher odds of non-primary first contact (aOR 4.68, 95% CI 1.24–30.71). Given the wide CI and multiple comparisons, this estimate is imprecise and should be interpreted cautiously.” | We rewrote the paragraph mentioning the association and the wide range of 95% confidence interval as limits in the interpretation of the results. | Page 9, 12, 13 |
| | L218 (witchcraft aOR=2.35, CI 1.06–5.45, p=0.04)<br><br>Issue: Same concerns; maintain associative language.<br><br>Fix (append): “A similar association was observed for witchcraft attributions (aOR 2.35, 95% CI 1.06–5.45).” | We rewrote the paragraph mentioning an association language, with the inclusion of the association of witchcraft attribution in the first sentence in the comparison. | Page 9 |
