# Supplementary material for: Beliefs, referrals, and mental healthcare pathways in the Eastern Democratic Republic of Congo
Source: PLOS Glob Public Health. 2026 Jul 2;6(7):e0006715. doi: 10.1371/journal.pgph.0006715 (PMC13327247; doi:10.1371/journal.pgph.0006715)
Supplement: Attachment — Submitted filename: responses to reviewers 28.2.2026.pdf [file pgph.0006715.s005.pdf]

Manuscript ID: PGPH-D-25-01441R1

Manuscript title: Beliefs, Referrals, and Mental Healthcare Pathways in the Eastern Democratic Republic of Congo

PLOS Global Public Health

RE: Corrections in response to reviewers' comments

Dear Editor

Greetings

Thank you for considering our manuscript and for the comments to make it perfect.

Please find in the table below the responses to reviewer and editor's questions and we hope that the current version meets the expectations of the reviewer and editorial teams.

Number	Questions from reviewer	Answer from author	Page
Reviewer #1			
A.General comments			
1	I think this paper has potential, but it needs editorial work to correct obvious errors. In addition, there are some substantive issues that need to be addressed or made more clearly:	We thank the reviewer for this comment. The manuscript has undergone careful editorial revision to correct grammatical errors, improve clarity, and address typographical inconsistencies throughout the text.	The whole manuscript
2	In some sections, the authors include community health workers under PHC, while in others, they contrast psychiatric centers against the PHC system. This can occasionally make it unclear if a "Psychiatric Hospital" is being treated as part of the formal medical system or as a separate entity that patients "self-refer" to because they don't trust the PHC.	We thank the reviewer for this important observation by specifying that psychiatric hospitals are not part of PHC. This is since in the DRC health system, primary health care (PHC) comprises health centers, general referral hospitals, and community health workers operating within defined health zones. Specialized psychiatric hospitals constitute a higher level of care within the formal health system and	Whole manuscript, especially in "method section"

		typically receive referrals from PHC facilities.	
	You could more strongly articulate what I think is an important contribution of this study: The current medical system in the DRC is bypassed because it fails to account for the cultural determinants of health. The authors draw on the global literature to show that, while the DRC's situation is extreme due to war and civil unrest, the solution—collaborative care between religious and medical leaders—is an evidence-based strategy that has been successfully used elsewhere. In addition, these points could be reinforced in the discussion and perhaps listed under a heading of "Late Entry into Care."	This has been taken into consideration in the discussion section, abstract and conclusions.	Pages 2, 14 & 15
	The high percentage of recurrent or progressive cases suggests that patients in Eastern DRC may only reach psychiatric hospitals once their condition has become chronic or severe. This likely correlates with the authors' earlier point that patients first spend months or years navigating religious or traditional pathways. Is that correct?	We thank the reviewer for the deep comprehension. This has been taken into consideration.	Page 13
	If 51% of participants were already attending for follow-up or refills, but were self-referred, it indicates a systemic disconnect. The Primary Healthcare (PHC) system is not just being bypassed for initial diagnosis; it is being bypassed for the long-term management of chronic disorders.	We reviewed the manuscript as needed by the reviewers	Page 14

	The authors should explicitly discuss whether the high relapse rate (69%) is a result of patients abandoning medical treatment in favor of religious centers during periods of remission, as hinted at by the 14.29% who returned to religious centers for their second contact.	This has been taken into consideration in the discussion section	13
	I believe citing the work of Eagle, Parker and Asobee (2025) could help strengthen your argument, they did a study of the beliefs of charismatic vs. non-charismatic Protestant pastors on the etiology and treatment of mental illness. They showed that many pastors held an integrated view (i.e. endorsed both medical and supernatural origins and treatments for mental illness.	We have added a citation to Eagle et al. (2025), which examines the influence of worship practices on pastors' beliefs about the causes and treatment of depression among Revivalist and Protestant pastors in the DRC.	Page 13
	Line 58: What does the 20-50% prevalence refer to: PTSD and depression combined or each have this range of prevalences? Also, am I right that you are saying that while 20% to 50% of the population may actually suffer from PTSD or depression due to the conflict, only a tiny fraction (1.9% to 2.5%) is actually being recorded or identified within the Primary Healthcare (PHC) facilities? If so, I would prefer it if you could make that point more clearly. It is an important fact to highlight.	We thank the reviewer for this important observation. Indeed, 20–50% refer to the population prevalence of depression and posttraumatic disorder according to existing findings, whereas the few studies in primary health care conducted in one of the health zones (Lubero health zone) in North Kivu suggest that 1.9% to 2.5% are being recorded or identified within the Primary Healthcare (PHC) facilities. Then, we highlighted this point more clearly in the background..	Page 13
Line 77	Line 77: “war torn” not “war tone”.	Thank you. The word “tone” has been replaced by “torn” in the manuscript	The whole manuscript
	(e.g. Lines 130-132 and Lines 153-158. There are repeated sentences)	This has been corrected.	Page 4

	This manuscript could benefit from comprehensive copy editing and improvement in the usage of English language.	We have reviewed the whole manuscript as needed by the reviewers	Whole manuscript
60 "In total, 47.3% of patients had their first care contact..."	Missing a descriptor. Likely should be: "had their first care contact at a psychiatric center..."	We thank the reviewer for the important observation. The clause has been replaced by the corrected one.	Page 2
66 "...having multiples episodes..."	Should be "multiple episodes" (remove the 's').	We thank the reviewer for the important observation. The clause has been replaced by the corrected one.	Page 10
108 "...majority of individuals..."	Missing article: "the majority of individuals..."	We thank the reviewer for the important observation. The clause has been replaced by the corrected one.	Page 1
112 "...seek for help..."	Delete "for": "...seek help from religious leaders..."	We thank the reviewer for the important observation. The clause has been replaced by the corrected one.	Page 14
114 "...disease genesis..."	Better as: "...the genesis of disease..." or "...disease etiology..."	We thank the reviewer for the important observation. The clause has been replaced by the corrected one.	Page 3
182 "...review the medical records... then obtained..."	Tense inconsistency: "...then obtain..." or "...reviewed... then obtained."	We thank the reviewer for the important observation. The clause has been replaced by the corrected one.	Page 4
183 "We did consecutive sampling techniques..."	Better as: "We utilized consecutive sampling techniques..."	We thank the reviewer for the important observation. The clause has been replaced by the corrected one.	Page 4
201 "...In regard with n religious orientation..."	Should be "In regard to religious orientation..."	We thank the reviewer for the important observation. The clause has been replaced by the corrected one.	Page 4

218 "...both possible and acceptable..."	Likely missing a word: "...both a possible and acceptable method..."	We thank the reviewer for the important observation. The clause has been replaced by the corrected one.	Page 5
252 "...management of mental health challenges..."	Missing a word: "...play a central role..."	We thank the reviewer for the important observation. The clause has been replaced by the corrected one.	Page 13
254 "This findings are in line..."	Should be "These findings are in line..."	We thank the reviewer for the important observation. The clause has been replaced by the corrected one.	Page 13-14
254 "...seek for care..."	Delete "for": "...seek care..."	We thank the reviewer for the important observation. The clause has been replaced by the corrected one.	Page 14
line 139	what is meant by a "God servant"? A religious leader/pastor? That would be a better term to use.	The term "religious center has been replaced with "religious leader" throughout the manuscript, denoting priests, pastors, and other faith leaders whom participants consulted.	Whole manuscript
Line 144	RStudio is an IDE. Please cite the version of R used in these analyses.	We thank the reviewer for this clarification. We have revised the method section to specify the version of R used for the analysis. Statistical analyses were conducted using R version 4.3.2 (R Foundation for Statistical Computing, Vienna, Australia) within the RStudio IDE.	Page 5
Table 2	Should be formatted to match the standards for a demographics table – reference other public health publications. Typically, this is Table 1 in a manuscript. I would suggest eliminating the current Table 1 and placing it in an	We thank the reviewer for this important observation. The term "religious center has been replaced with "religious leader" throughout the manuscript, denoting priests, pastors,	Whole manuscript

	Appendix or supplement. It is not vital to the analysis.	and other faith leaders whom participants consulted.	
Line 178	How was the relapse rate calculated? That is not clear. The manuscript claims a relapse rate of 69.45%. This appears to be a simple subtraction of the "First acute episode" percentage (30.45%) from 100%. This is a potentially misleading use of the term "relapse rate" in a cross-sectional study; it simply describes the proportion of the sample experiencing a non-first episode.	Thank you for the important observation. We have replaced "replaced rate" by "the proportion of the sample experiencing a non-first episode"	Page 6
Table 3	should read "God's punishment".	Thank you for the observation. "God's punishment" has been replaced by "Divine punishment"	Whole manuscript
Line 181 and following	More could be done to clarify what the figures are indicating.	Thank you for this clarification request. A description related to figures has been integrated in the paragraph.	Page 9
	And do you mean that 1/3 first consulted a religious center?	Yes, we mean that one-third sought help from a religious leader.	
	What are these religious centers?	To avoid ambiguity, we have replaced the term "religious center", which referred to places where persons sought help, with "religious leader" throughout the manuscript, referring to priests, pastors, and other faith leaders consulted for support.	Whole manuscript
	I don't understand the "God servant" language used in these figures, please clarify.	We thank the reviewer for pointing this out. We have therefore replaced it throughout the manuscript with the term "religious leader" to ensure clarity and	Figure 1-4

		consistency with academic terminology	
	The caption for the figure says Figure 1, but there are actually 4 figures.	We thank the reviewer for noting this oversight. The redundant caption has been removed, and the figure legends are provided below each figure	Figure 1-4
Table 4	I think there might be an error. PHC system is listed twice, shouldn't one be non-PHC?	This has been taken into consideration	
Table 5	I have concerns about the width of the confidence intervals, these suggest instability (i.e. lack of sufficient sample size) in the regression models. Consider coarsening the groups being compared to improve the stability of the estimates. Some categories (e.g. None for first contact, have a n=8 cell size) – that is likely too small a category to include. Also, for Nones there is no coverage in some cells (e.g. second contact for mental health being a PHC), which again makes coefficients impossible to estimate.	We have reviewed as needed by the reviewer by including the sentence "The width of the confidence intervals does require caution, as it suggests a sufficient sample size is lacking, which could affect the estimated correlations analyzed through the regression models" in the study limitations.	Page 15
	Are these regressions clustered by health care centers? Please clarify. If so, provide ICCs. Fit statistics are not reported for the models – they should be.	We have reviewed as needed by the reviewers	Page 5
	Several entries in this table use "NS" (Not Significant) or display the "aOR" and "P" values in a way that breaks the table alignment, making some rows difficult to read.	This ha been taken into consideration	Table3
Line 209	The word "unfortunately" could be removed.	This has been taken into consideration	
