# Supplementary material for: Beliefs, referrals, and mental healthcare pathways in the Eastern Democratic Republic of Congo
Source: PLOS Glob Public Health. 2026 Jul 2;6(7):e0006715. doi: 10.1371/journal.pgph.0006715 (PMC13327247; doi:10.1371/journal.pgph.0006715)
Supplement: Attachment — Submitted filename: Responses to reviewers 2.pdf [file pgph.0006715.s006.pdf]

Manuscript ID: PGPH-D-25-01441R2

Manuscript title: Beliefs, Referrals, and Mental Healthcare Pathways in the Eastern Democratic Republic of Congo
PLOS Global Public Health

RE: Corrections in response to reviewers' comments

Dear Editor Greetings
Thank you for considering our manuscript and for the comments to make it perfect. Please find in the table below the responses to reviewer and editor's questions and we hope that the current version meets the expectations of the reviewer and editorial teams.

S/N	Questions from reviewer	Answer from author	Page
1	Previous reviews of the manuscript seem to focus on the needed procedural clarification and clarity of some explanations and possible connectiveness of the psychiatric , religious, supernatural and medical issues. The statistical clarity overall is lacking despite the section on the Data processing and analysis plan. What about the ordering of type of contact if the numbers are sufficient to do so?	We thank the reviewer for this important comment. We have clarified in the method section that the referral pathway was assessed by recording the sequence of providers consulted before reaching psychiatric services, specifically the first, second, third, and fourth or subsequent points of contact with type of provider for each contact (community health worker, religious leader, natural healer, primary health care facility, psychiatric hospital, or unknown). We have also revised the appendix to explicitly present these ordered points of contact and the corresponding response categories, to better describe how the pathway sequence was captured.	Pg 5 Line 164-169
2	I can't open the 'sav' file if the pathway is any help.	The dataset has been resubmitted as an excel worksheet.	Appendix 2
3	The analysis is not clearly presented as well. They used a hierarchical separate binary logistic regression model by fitting for each contact	We thank the reviewer for highlighting the lack of clarity in the description of our regression analysis. In the revised manuscript, we clarified that we performed separate stage-specific binary logistic regression models for the first, second, third, and fourth points of contact, with the outcome dichotomized as PHC versus non-	Pg Line 180-199

	stage, to identify factors associated with the first, second, third, and fourth point of contact, all dichotomized into the two categories (PHC system and non-PHC system), recognizing first contact motivations may differ from those guiding later decisions. This is totally confusing and needs expansion.	PHC contact at each stage. We also clarified that cluster-robust standard errors at the hospital level were applied to account for within-hospital clustering. The term “hierarchical” was removed because it was misleading, as the models were not multilevel models and did not account for repeated within-person contacts longitudinally. We further explained that analyses at each stage were restricted to participants who had reached that stage, and that the purpose of separate models was to identify whether determinants differed across successive care-seeking contacts.	
4	If this is Table 2 (Predictors of seeking mental health services in non-primary health care facilities) then the affect of the ordering is certainly not clear for either the PHC or NPHC data.	We thank the reviewer for this observation. We have clarified that the dependent variable at each stage was coded as 1 = non-PHC contact and 0 = PHC contact in the table note. We also revised the table title to indicate that the results represent stage-specific predictors of seeking care in non-PHC versus PHC settings across the point of contact in seeking mental health care. In addition, a table note was added to clarify that an aOR greater than 1 indicates increased odds of non-PHC care-seeking, while an aOR less than 1 indicates decreased odds of seeking care in non-PHC setting. These revisions improve the interpretation of the ordering of care pathways across successive points of contact.	Pg 11
5	Also are the numbers sufficient for the variables or confounders considered in the models. What exactly is the content of the predictors in the models? The limitations in the ‘Discussion’ may need expansion to explain this.	We thank the reviewer for this important observation. We have clarified in the Methods section the exact predictors included in the multivariable models, namely sociodemographic factors (age, sex, marital status, employment), belief-related factors (religious affiliation, religiosity, perceived cause of illness), and clinical factors (admission mode, recurrence of symptoms, and family history of mental disorders), selected based on theoretical relevance and prior literature.<br>We also expanded the limitations section to acknowledge that the number of predictors included in the regression models may have been high relative to the number of outcome events at later contact stages. This may have led to overfitting, unstable estimates, sparse-data	Pg<br>Line 180-199

		bias, and wide confidence intervals, limiting the precision of some adjusted odds ratios. We now explicitly state that findings from later-stage models should be interpreted cautiously and that larger studies are needed to confirm these associations.	
6	The entire statistical presentation needs a thorough edit for clarity.	We have clarified the entire statistical presentation	Pg<br>Line 180-199
