# Supplementary material for: Beliefs, referrals, and mental healthcare pathways in the Eastern Democratic Republic of Congo
Source: PLOS Glob Public Health. 2026 Jul 2;6(7):e0006715. doi: 10.1371/journal.pgph.0006715 (PMC13327247; doi:10.1371/journal.pgph.0006715)
Supplement: Attachment — Submitted filename: PlosGHDRC.docx [file pgph.0006715.s004.docx]

The paper argues that in conflict-affected regions of the Eastern Democratic Republic of Congo (DRC), primary healthcare (PHC) is significantly underutilized for mental health needs, with patients instead favoring self-referral to psychiatric hospitals or religious centers. The authors contend that this behavior is driven by strong cultural and religious beliefs—specifically attributing mental illness to witchcraft, supernatural powers, or divine punishment—and by the lack of integrated mental health services within the PHC system. The study advocates for a collaborative model between religious leaders and medical professionals to improve care pathways.

I think this paper has potential, but it needs editorial work to correct obvious errors. In addition, there are some substantive issues that need to be addressed or made more clearly:

In some sections, the authors include community health workers under PHC, while in others, they contrast psychiatric centers against the PHC system. This can occasionally make it unclear if a "Psychiatric Hospital" is being treated as part of the formal medical system or as a separate entity that patients "self-refer" to because they don't trust the PHC.

You could more strongly articulate what I think is an important contribution of this study: The current medical system in the DRC is bypassed because it fails to account for the cultural determinants of health. The authors draw on the global literature to show that, while the DRC's situation is extreme due to war and civil unrest, the solution—collaborative care between religious and medical leaders—is an evidence-based strategy that has been successfully used elsewhere. In addition, these points could be reinforced in the discussion and perhaps listed under a heading of “Late Entry into Care.”

The high percentage of recurrent or progressive cases suggests that patients in Eastern DRC may only reach psychiatric hospitals once their condition has become chronic or severe. This likely correlates with the authors' earlier point that patients first spend months or years navigating religious or traditional pathways. Is that correct?

If 51% of participants were already attending for follow-up or refills, but  were self-referred, it indicates a systemic disconnect. The Primary Healthcare (PHC) system is not just being bypassed for initial diagnosis; it is being bypassed for the long-term management of chronic disorders.

The authors should explicitly discuss whether the high relapse rate (69%) is a result of patients abandoning medical treatment in favor of religious centers during periods of remission, as hinted at by the 14.29% who returned to religious centers for their second contact.

I believe citing the work of Eagle, Parker and Asobee (2025) could help strengthen your argument, they did a study of the beliefs of charismatic vs. non-charismatic Protestant pastors on the etiology and treatment of mental illness. They showed that many pastors held an integrated view (i.e. endorsed both medical and supernatural origins and treatments for mental illness.

Line 58: What does the 20-50% prevalence refer to: PTSD and depression combined or each have this range of prevalences? Also, am I right that you are saying that while 20% to 50% of the population may actually suffer from PTSD or depression due to the conflict , only a tiny fraction (1.9% to 2.5%) is actually being recorded or identified within the Primary Healthcare (PHC) facilities? If so, I would prefer it if you could make that point more clearly. It is an important fact to highlight.

Line 77: “war torn” not “war tone”. There a number of careless errors throughout this manuscript (e.g. Lines 130-132 and Lines 153-158. There are repeated sentences) and this manuscript could benefit from comprehensive copy editing and improvement in the usage of English language. Here is a list of errors that I found in the manuscript:

FYI, these are other grammatical errors in the manuscript.

| Line Number | Error | Suggestion |
| --- | --- | --- |
| 60 | "In total, 47.3% of patients had their first care contact..." | Missing a descriptor. Likely should be: "had their first care contact at a psychiatric center..." |
| 66 | "...having multiples episodes..." | Should be "multiple episodes" (remove the 's'). |
| 108 | "...majority of individuals..." | Missing article: "the majority of individuals..." |
| 112 | "...seek for help..." | Delete "for": "...seek help from religious leaders..." |
| 114 | "...disease genesis..." | Better as: "...the genesis of disease..." or "...disease etiology..." |
| 182 | "...review the medical records... then obtained..." | Tense inconsistency: "...then obtain..." or "...reviewed... then obtained." |
| 183 | "We did consecutive sampling techniques..." | Better as: "We utilized consecutive sampling techniques..." |
| 201 | "...In regard with n religious orientation..." | Should be "In regard to religious orientation..." |
| 218 | "...both possible and acceptable..." | Likely missing a word: "...both a possible and acceptable method..." |
| 252 | "...management of mental health challenges..." | Missing a word: "...play a central role..." |
| 254 | "This findings are in line..." | Should be "These findings are in line..." |
| 254 | "...seek for care..." | Delete "for": "...seek care..." |

On line 139, what is meant by a “God servant”? A religious leader/pastor? That would be a better term to use.

Line 144, RStudio is an IDE. Please cite the version of R used in these analyses.

Table 2 should be formatted to match the standards for a demographics table – reference other public health publications. Typically, this is Table 1 in a manuscript. I would suggest eliminating the current Table 1 and placing it in an Appendix or supplement. It is not vital to the analysis.

Line 178: How was the relapse rate calculated? That is not clear. The manuscript claims a relapse rate of 69.45%. This appears to be a simple subtraction of the "First acute episode" percentage (30.45%) from 100%. This is a potentially misleading use of the term "relapse rate" in a cross-sectional study; it simply describes the proportion of the sample experiencing a non-first episode.

Table 3 should read “God’s punishment”.

Lines 181 and following: More could be done to clarify what the figures are indicating. And do you mean that 1/3 first consulted a religious center? What are these religious centers? I don’t understand the “God servant” language used in these figures, please clarify. The caption for the figure says Figure 1, but there are actually 4 figures.

Table 4, I think there might be an error. PHC system is listed twice, shouldn’t one be non-PHC?

Table 5: I have concerns about the width of the confidence intervals, these suggest instability (i.e. lack of sufficient sample size) in the regression models. Consider coarsening the groups being compared to improve the stability of the estimates. Some categories (e.g. None for first contact, have a n=8 cell size) – that is likely too small a category to include. Also, for Nones there is no coverage in some cells (e.g. second contact for mental health being a PHC), which again makes coefficients impossible to estimate. Are these regressions clustered by health care center? Please clarify. If so, provide ICCs. Fit statistics are not reported for the models – they should be. Several entries in this table use "NS" (Not Significant) or display the "aOR" and "P" values in a way that breaks the table alignment, making some rows difficult to read.

Line 209: the word “unfortunately” could be removed.
