# Supplementary material for: Beliefs, referrals, and mental healthcare pathways in the Eastern Democratic Republic of Congo
Source: PLOS Glob Public Health. 2026 Jul 2;6(7):e0006715. doi: 10.1371/journal.pgph.0006715 (PMC13327247; doi:10.1371/journal.pgph.0006715)
Supplement: Peer review history

# Peer review: Decision Letter 0

**DOI:** 10.1371/journal.pgph.0006715.r001

**Authors:** Collins Asweto

2 Sep 2025

PGPH-D-25-01441

Entry Points to Mental Healthcare: The Influence of Beliefs and Referrals on the Use of the Primary Healthcare System in North Kivu Province, Eastern Democratic Republic of Congo

PLOS Global Public Health

Dear Bives,

Thank you for submitting your manuscript to PLOS Global Public Health. After careful consideration, we feel that it has merit but does not fully meet PLOS Global Public Health’s publication criteria as it currently stands. Therefore, we invite you to submit a revised version of the manuscript that addresses the points raised during the review process.

Please submit your revised manuscript by 1st November, 2025. If you will need more time than this to complete your revisions, please reply to this message or contact the journal office at globalpubhealth@plos.org. When you're ready to submit your revision, log on to https://www.editorialmanager.com/pgph/ and select the 'Submissions Needing Revision' folder to locate your manuscript file.

Please include the following items when submitting your revised manuscript:

- A rebuttal letter that responds to each point raised by the editor and reviewer(s). You should upload this letter as a separate file labeled 'Response to Reviewers'.
- A marked-up copy of your manuscript that highlights changes made to the original version. You should upload this as a separate file labeled 'Revised Manuscript with Track Changes'.
- An unmarked version of your revised paper without tracked changes. You should upload this as a separate file labeled 'Manuscript'.

Guidelines for resubmitting your figure files are available below the reviewer comments at the end of this letter.

We look forward to receiving your revised manuscript.

Kind regards,

Collins Otieno Asweto, PhD

Academic Editor

PLOS Global Public Health

Journal Requirements:

1. Please update your online Competing Interests statement. If you have no competing interests to declare, please state: “The authors have declared that no competing interests exist.”

2. We note that you have indicated that there are restrictions to data sharing for this study. For studies involving human research participant data or other sensitive data, we encourage authors to share de-identified or anonymized data. However, when data cannot be publicly shared for ethical reasons, we allow authors to make their data sets available upon request. For information on unacceptable data access restrictions, please see http://journals.plos.org/globalpublichealth/s/data-availability#loc-unacceptable-data-access-restrictions.

Before we proceed with your manuscript, please address the following prompts:

a) If there are ethical or legal restrictions on sharing a de-identified data set, please explain them in detail (e.g., data contain potentially identifying or sensitive patient information, data are owned by a third-party organization, etc.) and who has imposed them (e.g., a Research Ethics Committee or Institutional Review Board, etc.). Please also provide contact information for a data access committee, ethics committee, or other institutional body to which data requests may be sent.

b) If there are no restrictions, please upload the minimal anonymized data set necessary to replicate your study findings to a stable, public repository and provide us with the relevant URLs, DOIs, or accession numbers. Please see http://www.bmj.com/content/340/bmj.c181.long for guidelines on how to de-identify and prepare clinical data for publication. For a list of recommended repositories, please see https://journals.plos.org/globalpublichealth/s/recommended-repositories. You also have the option of uploading the data as Supporting Information files, but we would recommend depositing data directly to a data repository if possible.

Please update your Data Availability statement in the submission form accordingly.

3. If the reviewer comments include a recommendation to cite specific previously published works, please review and evaluate these publications to determine whether they are relevant and should be cited. There is no requirement to cite these works unless the editor has indicated otherwise.

4. Please review your reference list to ensure that it is complete and correct. If you have cited papers that have been retracted, please include the rationale for doing so in the manuscript text, or remove these references and replace them with relevant current references. Any changes to the reference list should be mentioned in the rebuttal letter that accompanies your revised manuscript. If you need to cite a retracted article, indicate the article’s retracted status in the References list and also include a citation and full reference for the retraction notice.

Additional Editor Comments (if provided):

**Comments to the Author**

1. Does this manuscript meet PLOS Global Public Health’s publication criteria? Is the manuscript technically sound, and do the data support the conclusions? The manuscript must describe methodologically and ethically rigorous research with conclusions that are appropriately drawn based on the data presented.

Reviewer #1: Yes

Reviewer #2: Yes

Reviewer #3: Yes

Reviewer #4: Yes

Reviewer #5: Partly

**********

2. Has the statistical analysis been performed appropriately and rigorously?

Reviewer #1: Yes

Reviewer #2: Yes

Reviewer #3: Yes

Reviewer #4: Yes

Reviewer #5: No

**********

3. Have the authors made all data underlying the findings in their manuscript fully available (please refer to the Data Availability Statement at the start of the manuscript PDF file)?

The PLOS Data policy requires authors to make all data underlying the findings described in their manuscript fully available without restriction, with rare exception. The data should be provided as part of the manuscript or its supporting information, or deposited to a public repository. For example, in addition to summary statistics, the data points behind means, medians and variance measures should be available. If there are restrictions on publicly sharing data—e.g. participant privacy or use of data from a third party—those must be specified.

Reviewer #1: No

Reviewer #2: Yes

Reviewer #3: Yes

Reviewer #4: Yes

Reviewer #5: Yes

**********

4. Is the manuscript presented in an intelligible fashion and written in standard English?

PLOS Global Public Health does not copyedit accepted manuscripts, so the language in submitted articles must be clear, correct, and unambiguous. Any typographical or grammatical errors should be corrected at revision, so please note any specific errors here.

Reviewer #1: No

Reviewer #2: Yes

Reviewer #3: Yes

Reviewer #4: Yes

Reviewer #5: Yes

**********

5. Review Comments to the Author

Please use the space provided to explain your answers to the questions above. You may also include additional comments for the author, including concerns about dual publication, research ethics, or publication ethics. (Please upload your review as an attachment if it exceeds 20,000 characters)

Reviewer #1: Thank you for submitting this manuscript, makes significant contribution in exploring mental healthcare pathways in a conflict-affected region, addressing an important research gap with original data from a challenging context. While the study is methodologically sound and offers valuable insights, several aspects—particularly clarity of language, structure, and adherence to reporting and data-sharing standards—require improvement. Here below are issues that need to be addressed: Summary of Key Issues

Data Availability: Per PLOS policy, deposit anonymized data in a public repository or provide a valid reason for exemption.

Section-by-Section Feedback

A. Title &

1. Consider shortening the title for clarity, e.g., ‘Beliefs, Referrals, and Mental Healthcare Pathways in Eastern DRC’ to make it more concise while retaining the main elements.

B. Abstract Paragraph 1 (lines 27–49)

2. Issue: The abstract is lengthy with some redundancy (e.g., repeating “psychiatric hospital” multiple times).

Suggestion: remove redundancy, streamline background; focus on key statistics, main predictors, and implications. Include important statistical results (aORs, p-values) for stronger impact. Ensure all abbreviations (e.g., PHC) are defined on first use.

C. Introduction(lines 52–57)

3. Correct grammar in paragraph 1 (“play” → “plays”), and ensure consistent tense usage.

4. Shorten long sentences in paragraph 3 for better readability.

5. End the introduction with a one-sentence, clearly stated research objective/hypothesis.

6. lines 66–77

Issue: Long sentences with multiple clauses reduce clarity.

Suggestion: Break into shorter sentences for readability.

7. Lines 96–102

Issue: Study aim is stated but not as a clear, concise research objective.

Suggestion: End the Introduction with a specific, one-sentence statement of objectives or hypothesis.

D. Methods

6. Rewrite hospital inclusion description for clarity (paragraph 2).

7. Simplify questionnaire description (paragraph 4).

8. Justify or cite categorization of religiosity (paragraph 6).

9. Specify all variables used in regression and cite literature for selection (paragraph 9).

10.

8. Lines 105–118; Study Setting

Issue: Minor grammar and clarity issues (“resulting in Polycliniques…” is awkward).

Suggestion: Rewrite for clarity, e.g., ‘The selected hospitals included… These facilities met inclusion criteria of…’.

9. lines 131–143; Data Collection

Issue: Phrase ‘never been published’ in describing questionnaire is redundant.

Suggestion: Simply state ‘a structured questionnaire developed for this study.’

10. lines 153–158; Religiosity Measures

Issue: Definition of ‘very religious’ as ‘more than five days a week’ may need referencing or justification.

Suggestion: Cite supporting literature for these cut-offs or note they were based on pilot testing.

11. lines 172–182; Statistical Analyses

Issue: Variables included in regression are said to be ‘chosen based on existing studies’ without listing them fully.

Suggestion: Specify variables and cite relevant studies for transparency.

E. Results

12. lines 194–199; Clinical Characteristics

Issue: Reported relapse rate (69.5%) is important but not discussed in results narrative.

Suggestion: Briefly contextualize its significance in the results section.

13. Tables 2–4

Issue: Inconsistent p-value and CI formatting across tables/figures.

Suggestion: Standardize format per PLOS style (e.g., p=0.05, 95% CI [x–y]) in all tables.

F. Discussion

14. lines 229–237

Issue: Repeats content from abstract nearly verbatim.

Suggestion: Focus on interpreting findings rather than restating them.

15. lines 268–277); Integration Recommendation

Issue: Long sentence chains make the recommendation difficult to follow.

Suggestion: Split into separate sentences: one for holistic model advocacy, one for integration, one for collaboration mechanisms.

16. Lines 278–291; Policy Implications

Issue: Important points on stigma and cultural beliefs, but needs references to back claims (e.g., ‘health workers… do not have adequate training’).

Suggestion: Add citations or state that this is based on study findings.

G. Limitations

17. Lines 292–305

Issue: ‘Quantitative approached’ correct ‘approach’ to ‘approach’, clarify whether 404 is truly low sample size for cross-sectional design

Suggestion: Revise wording to reflect actual limitation and avoid misleading reader.

H. Conclusion

18. Lines 306–312

Issue: Restates results without linking back to broader literature or practical implications in depth.

Suggestion: End with a strong recommendation for policy/practice based on findings, i.e. Strengthen conclusion with specific, actionable recommendations.

I. Other general Points.

19. Language: Throughout, minor grammar and syntax issues need correction (subject–verb agreement, tense consistency). Recommend professional English editing.

20. Reporting Guidelines: Add explicit statement in Methods noting adherence to STROBE checklist for cross-sectional studies.

21. Data Availability: PLOS encourages depositing anonymized data in a public repository rather than ‘available on request.’

22. Reference List: Check for over-reliance on the authors’ own work and ensure balanced citation of independent studies.

Reviewer #2: Reviewer Report

Manuscript title: Entry Points to Mental Healthcare: The Influence of Beliefs and Referrals on the Use of the Primary Healthcare System in North Kivu Province, Eastern Democratic Republic of Congo

Manuscript number: PGPH-D-25-01441

Article Type: Research Article

Overall Assessment

This manuscript addresses an important and under-researched topic—pathways to mental health care in conflict-affected settings—using data from psychiatric hospitals in Eastern DRC. The focus on religious beliefs, self-referral patterns, and primary healthcare underutilization is relevant for health systems research and aligns with PLOS Global Public Health’s scope.

However, the paper has substantial weaknesses in writing clarity, methodological rigor, result presentation, and interpretation of findings. There are contradictions between reported numbers, unclear definitions of key terms, insufficient statistical detail, and overgeneralized conclusions that are not fully supported by the study design. The limitations section underplays key biases, and the recommendations are broad without a clear evidence-based implementation framework.

In its current form, the manuscript requires major revision before it can be considered for publication.

Major Comments

1. Writing Clarity and Structure

The manuscript contains multiple grammatical errors, awkward phrasing, and inconsistent terminology (e.g., “war-tone” vs “war-torn”; “non-conventional hospitals” vs “non-primary healthcare hospitals” vs “psychiatric hospitals”).

Many sentences are overly long and repetitive, reducing clarity for an international audience. The Abstract and Discussion in particular repeat the same statistics without adding new interpretation.

Clearer definitions are needed for “non-conventional hospital,” “primary healthcare hospital,” and “psychiatric hospital” early in the Methods to avoid confusion.

2. Contradictions in Findings

Self-referral was reported as 89.2% (Results) and also described as 47.3% first contact with psychiatric hospitals (Figure 1), which appears inconsistent. Authors should reconcile or explain this discrepancy.

In the Discussion, “one in three” patients seeking religious care after hospital treatment is highlighted as a major finding, but this pattern is not clearly supported by the presented data tables.

The proportion attributing illness to “God’s punishment” (12.85%) is comparatively small, yet it is discussed as a dominant explanatory factor without proportional emphasis on witchcraft (32.67%) or natural punishment (29.95%).

3. Methodological Rigor

Sampling bias – The sample consists only of psychiatric hospital patients, excluding individuals who never access these facilities. This likely overestimates the proportion of non-primary healthcare pathways.

Instrument validation – The survey tool was self-developed; while piloted, no psychometric properties (e.g., reliability coefficients, validity evidence) are provided.

Recall bias – Pathway-to-care histories rely entirely on self-report, which is particularly vulnerable to recall error in multi-contact health-seeking patterns. This is not discussed as a limitation.

Variable selection for regression – Factors “chosen based on existing studies” is too vague. Authors should specify model-building strategy and justify inclusion/exclusion.

4. Results Reporting

Inconsistent sample sizes: Table 2 reports n=411, but Methods specifies n=404.

Regression results have extremely wide confidence intervals (e.g., aOR=9.86, CI=1.35–86.82), indicating unstable estimates likely due to small subgroup counts—yet no cautionary interpretation is provided.

Missing model diagnostics (goodness-of-fit, pseudo-R²) and no check for multicollinearity between predictors.

Figure 1 lacks axis labels, clear legends, and an explanation of what “n” values represent at each stage.

5. Interpretation and Overgeneralization

The authors make causal-sounding claims (e.g., “beliefs increase the odds”) from cross-sectional association data without sufficient caution.

Recommendations to integrate religious leaders into formal care systems are not directly supported by intervention evidence from this study.

Conclusions generalize to “most patients with mental disorders in war-torn zones” despite only sampling those attending psychiatric hospitals.

6. Limitations

Important biases—recall bias, social desirability bias, and misclassification—are missing from the limitations section.

No discussion on the absence of socioeconomic variables (income, education) in regression models, despite their likely influence.

Missing data handling is not described.

Minor Comments

Abstract: Clarify the meaning of “non-conventional hospitals” and ensure percentages match those in Results.

Introduction: The review of literature is relevant but would benefit from more DRC-specific policy context (e.g., mhGAP integration efforts).

Methods: Specify how missing values were addressed and whether any data imputation was performed.

Tables: Include denominators for percentages and ensure all abbreviations (PHC, aOR) are defined in footnotes.

Discussion: Reduce repetition of numerical results already presented; focus on interpreting unexpected or contextually important findings.

Language: Correct typographical errors (“necessarily modifications” → “necessary modifications”, “tone” → “torn”).

References: Ensure consistent formatting according to PLOS style (some references are missing full journal details).

Recommendation

Major Revision – While the topic is important and the dataset potentially valuable, substantial rewriting, methodological clarification, and data reporting improvements are needed before the manuscript can be considered for acceptance.

Reviewer #3: Authors have chosen a legible topic that needs to get a high appreciatory. Introductory section may be further developed to add some more relevant literature. Implications of the study may galvanize other readers to follow same type of study.

Reviewer #4: Overall: Several sentences exceed 30 words; shortening would improve clarity. Consistent tense usage (past for methods/results, present for implications) should be ensured.

Abstract: Well-structured, but some sentences are long and could be more concise.

L28–31: Combine to avoid redundancy in stating the knowledge gap.

L34: “patients who attending” → “patients attending.”

L46–47: “due to such determinant” → “due to determinants such as.”

Introduction

L52: “Although primary health care play” → “plays.”

Good framing of cultural and systemic barriers; relevant literature cited.

L63–65: Sentence structure awkward; revise for clarity (“have not centered on people in war-torn settings or on patterns…”).

L78–81: Strong justification with prevalence data.

L87–94: Clear explanation of theoretical framework; well-linked to study aim.

L97–101: Slight grammatical issues; tighten for clarity. For example, “aimed to the patterns” → “aimed to examine the patterns.”

Methods

L106–113: Consider moving the rationale for hospital selection to a bulleted list for clarity.

Participant criteria clear; statistical reasoning for sample size appropriate.

L138–141: “Never been published” not necessary unless relevant for novelty claim.

Translation and piloting described well; however, specify inter-rater reliability or quality control in data collection.

Variables section is detailed; some long sentences could be split for readability.

Statistical analysis description appropriate; mention if model diagnostics were performed.

Results

Tables are informative; ensure consistent decimal formatting and labeling.

L195–199: Minor grammar issues (“relapse rate was 69.5%”).

Figure 1 reference is good; ensure high-quality, clearly labeled figures for publication.

L215–227: Well-presented predictors; however, Table 5 should be integrated in-text with more interpretation.

Discussion

L229–237: Nicely summarizes key findings upfront.

Well-linked to literature, but several sentences are long and contain multiple ideas (e.g., L239–246). Breaking these will improve flow.

Strong point on post-hospital religious center visits; valuable policy implication.

L268–277: Good integration of recommendations for collaborative models. Could emphasize feasibility and examples from similar contexts.

L278–291: Highlights cultural determinants effectively; could add a brief comment on stigma reduction strategies.

L292–305: Limitations are well-acknowledged; however, some phrasing is defensive (“may affect the responses of participants”). Instead, frame as neutral methodological constraint.

Could mention potential recall bias due to self-reported pathways.

Reviewer #5: The topic is important and the dataset is valuable, but the current manuscript overreaches the design and under‑specifies the analysis. With the reanalysis, clearer definitions/reporting, and toned‑down interpretation, it could become publishable.

Firstly the main claim of the study implies a causal association between beliefs and referrals and use of primary healthcare system and yet the study design is cross-sectional. This is Moreso given that the sampling method had no randomization and introduces potential bias. I would suggest that the authors consider a more conservative claim and do so consistently from title, abstract to discussion. Below are some specific observations of areas they can tighten

L230 (“we sought to determine the patterns and determinants…”)

Issue: “Determine” implies causality.

Possible Fix: “we sought to describe patterns and identify factors associated with pathways…”

“After treatment successes” implies efficacy and temporal sequence not established.

Fix (replace L235–237):

“Among those presenting to psychiatric hospitals, 359/402 (89.2%) reported self‑referral. Of those whose first contact was a hospital (n= [insert]), ~1/3 reported subsequently consulting a religious center. These self‑reported sequences are subject to recall error and do not establish treatment effectiveness.”

While the statistical approach is well suited to cross-sectional categorical data, the conclusions that are arrived at from the significance are faulty in that they establish causality. While multinomial logistic regression is appropriate for categorical outcomes, the analysis overlooks repeated measures, clustering by hospital, sparse data bias, and multiple comparisons. Without addressing these and clarifying definitions, results risk bias and overinterpretation. Re-analysis with robust, stage-aware methods and cautious interpretation is required.

Take care of repeated measures, the same participant may contribute data to multiple contact stages, but the model treats these as independent. This can underestimate standard errors. A stage-aware method (e.g., GEE or multilevel modelling with participant ID and hospital as clusters) would be more robust.

Possibly consider clustering by hospital, participants are nested within nine hospitals, which may share referral patterns or patient profiles. Ignoring this clustering risks overstating precision. Cluster-robust SEs or random effects are recommended.

L216–220

Unit of analysis and model not fully clear for stage‑specific outcomes; repeated measures likely (same person contributes multiple “contact points”), yet independence is implied.

This is an issue because it violated independence inflates Type I error and narrows CIs.

You may correct by stating the modeling approach and address dependence (GEE or mixed‑effects) and hospital clustering; clarify that stage models are conditional on reaching that stage.

My suggested way to correct is to add after L216

“We modeled stage‑specific outcomes conditional on participants having reached that stage. To account for repeated measures across stages and clustering by hospital, we used [GEE with logit link and exchangeable correlation / mixed‑effects logistic regression with random intercepts for participant and hospital]. Results are reported as adjusted odds ratios (aOR) with cluster‑robust 95% CIs.”

Below are some other very specific observations and suggestions on how the authors may strengthen the analysis

L217–218 (“God’s punishment” aOR=4.68, CI 1.24–30.71, p=0.05)

Issue: Very wide CI and borderline p; precision is low; multiple testing not acknowledged.

Why: Risk of false positives and sparse‑data bias.

Action: Flag imprecision; consider penalized methods (Firth) or category collapse if sparse.

You may replace sentence:

“Attributing challenges to God’s punishment was associated with higher odds of non‑primary first contact (aOR 4.68, 95% CI 1.24–30.71). Given the wide CI and multiple comparisons, this estimate is imprecise and should be interpreted cautiously.”

L218 (witchcraft aOR=2.35, CI 1.06–5.45, p=0.04)

Issue: Same concerns; maintain associative language.

Fix (append): “A similar association was observed for witchcraft attributions (aOR 2.35, 95% CI 1.06–5.45).”

**********

6. PLOS authors have the option to publish the peer review history of their article (what does this mean?). If published, this will include your full peer review and any attached files.

**Do you want your identity to be public for this peer review?**  If you choose “no”, your identity will remain anonymous but your review may still be made public.

For information about this choice, including consent withdrawal, please see our Privacy Policy.

Reviewer #1: No

Reviewer #2: No

Reviewer #3: **Yes:**  Dr. Mohammad Ismail Bhuiyan

Reviewer #4: No

Reviewer #5: **Yes:**  Efison Dhodho

**********

[NOTE: If reviewer comments were submitted as an attachment file, they will be attached to this email and accessible via the submission site. Please log into your account, locate the manuscript record, and check for the action link "View Attachments". If this link does not appear, there are no attachment files.]

While revising your submission, please upload your figure files to the Preflight Analysis and Conversion Engine (PACE) digital diagnostic tool, https://pacev2.apexcovantage.com/. PACE helps ensure that figures meet PLOS requirements. To use PACE, you must first register as a user. Registration is free. Then, login and navigate to the UPLOAD tab, where you will find detailed instructions on how to use the tool. If you encounter any issues or have any questions when using PACE, please email PLOS at figures@plos.org. Please note that Supporting Information files do not need this step.

# Author comment: Author response to Decision Letter 1

**DOI:** 10.1371/journal.pgph.0006715.r002

27 Oct 2025

Attachment Submitted filename: responses to reviewers 23.10.2025.pdf

# Peer review: Decision Letter 1

**DOI:** 10.1371/journal.pgph.0006715.r003

**Authors:** Collins Asweto, Helen Howard

4 Feb 2026

PGPH-D-25-01441R1

Beliefs, Referrals, and Mental Healthcare Pathways in the Eastern Democratic Republic of Congo

PLOS Global Public Health

Dear Dr. Mutume Nzanzu Vivalya,

Thank you for submitting your manuscript to PLOS Global Public Health. After careful consideration, we feel that it has merit but does not fully meet PLOS Global Public Health’s publication criteria as it currently stands. Therefore, we invite you to submit a revised version of the manuscript that addresses the points raised during the review process.

Please submit your revised manuscript by Mar 21 2026 11:59PM. If you will need more time than this to complete your revisions, please reply to this message or contact the journal office at globalpubhealth@plos.org. When you're ready to submit your revision, log on to https://www.editorialmanager.com/pgph/ and select the 'Submissions Needing Revision' folder to locate your manuscript file.

Please include the following items when submitting your revised manuscript:

- A letter that responds to each point raised by the editor and reviewer(s). You should upload this letter as a separate file labeled 'Response to Reviewers'.
- A marked-up copy of your manuscript that highlights changes made to the original version. You should upload this as a separate file labeled 'Revised Manuscript with Track Changes'.
- An unmarked version of your revised paper without tracked changes. You should upload this as a separate file labeled 'Manuscript'.

Guidelines for resubmitting your figure files are available below the reviewer comments at the end of this letter.

We look forward to receiving your revised manuscript.

Kind regards,

Helen Howard

Staff Editor

PLOS Global Public Health

Journal Requirements:

If the reviewer comments include a recommendation to cite specific previously published works, please review and evaluate these publications to determine whether they are relevant and should be cited. There is no requirement to cite these works unless the editor has indicated otherwise.

Additional Editor Comments (if provided):

[Note: HTML markup is below. Please do not edit.]

Reviewers' comments:

Reviewer's Responses to Questions

**Comments to the Author**

1. If the authors have adequately addressed your comments raised in a previous round of review and you feel that this manuscript is now acceptable for publication, you may indicate that here to bypass the “Comments to the Author” section, enter your conflict of interest statement in the “Confidential to Editor” section, and submit your "Accept" recommendation.

Reviewer #3: All comments have been addressed

Reviewer #6: All comments have been addressed

**********

2. Does this manuscript meet PLOS Global Public Health’s publication criteria? Is the manuscript technically sound, and do the data support the conclusions? The manuscript must describe methodologically and ethically rigorous research with conclusions that are appropriately drawn based on the data presented.

Reviewer #3: Yes

Reviewer #6: Yes

**********

3. Has the statistical analysis been performed appropriately and rigorously?

Reviewer #3: Yes

Reviewer #6: Yes

**********

4. Have the authors made all data underlying the findings in their manuscript fully available (please refer to the Data Availability Statement at the start of the manuscript PDF file)?

The PLOS Data policy requires authors to make all data underlying the findings described in their manuscript fully available without restriction, with rare exception. The data should be provided as part of the manuscript or its supporting information, or deposited to a public repository. For example, in addition to summary statistics, the data points behind means, medians and variance measures should be available. If there are restrictions on publicly sharing data—e.g. participant privacy or use of data from a third party—those must be specified.

Reviewer #3: Yes

Reviewer #6: No

**********

5. Is the manuscript presented in an intelligible fashion and written in standard English?

PLOS Global Public Health does not copyedit accepted manuscripts, so the language in submitted articles must be clear, correct, and unambiguous. Any typographical or grammatical errors should be corrected at revision, so please note any specific errors here.

Reviewer #3: Yes

Reviewer #6: No

**********

6. Review Comments to the Author

Please use the space provided to explain your answers to the questions above. You may also include additional comments for the author, including concerns about dual publication, research ethics, or publication ethics. (Please upload your review as an attachment if it exceeds 20,000 characters)

Reviewer #3: (No Response)

Reviewer #6: See attachment

**********

7. PLOS authors have the option to publish the peer review history of their article (what does this mean?). If published, this will include your full peer review and any attached files.

**Do you want your identity to be public for this peer review?**  If you choose “no”, your identity will remain anonymous but your review may still be made public.

For information about this choice, including consent withdrawal, please see our Privacy Policy.

Reviewer #3: **Yes:**  Dr Mohammad Ismail Bhuiyan

Reviewer #6: No

**********

[NOTE: If reviewer comments were submitted as an attachment file, they will be attached to this email and accessible via the submission site. Please log into your account, locate the manuscript record, and check for the action link "View Attachments". If this link does not appear, there are no attachment files.]

-->Figure Resubmissions:

While revising your submission, we strongly recommend that you use PLOS’s NAAS tool (https://ngplosjournals.pagemajik.ai/artanalysis) to test your figure files. NAAS can convert your figure files to the TIFF file type and meet basic requirements (such as print size, resolution), or provide you with a report on issues that do not meet our requirements and that NAAS cannot fix.

After uploading your figures to PLOS’s NAAS tool - https://ngplosjournals.pagemajik.ai/artanalysis, NAAS will process the files provided and display the results in the "Uploaded Files" section of the page as the processing is complete. If the uploaded figures meet our requirements (or NAAS is able to fix the files to meet our requirements), the figure will be marked as "fixed" above. If NAAS is unable to fix the files, a red "failed" label will appear above. When NAAS has confirmed that the figure files meet our requirements, please download the file via the download option, and include these NAAS processed figure files when submitting your revised manuscript.

Attachment Submitted filename: PlosGHDRC.docx

# Author comment: Author response to Decision Letter 2

**DOI:** 10.1371/journal.pgph.0006715.r004

28 Feb 2026

Attachment Submitted filename: responses to reviewers 28.2.2026.pdf

# Peer review: Decision Letter 2

**DOI:** 10.1371/journal.pgph.0006715.r005

**Authors:** Collins Asweto, Helen Howard, Alejandro Torrado Pacheco

6 Apr 2026

PGPH-D-25-01441R2

Beliefs, Referrals, and Mental Healthcare Pathways in the Eastern Democratic Republic of Congo

PLOS Global Public Health

Dear Dr. Mutume Nzanzu Vivalya,

Thank you for submitting your manuscript to PLOS Global Public Health. After careful consideration, we feel that it has merit but does not fully meet PLOS Global Public Health’s publication criteria as it currently stands. Therefore, we invite you to submit a revised version of the manuscript that addresses the points raised during the review process.

The manuscript has been evaluated by two reviewers. Reviewer 7 notes several significant issues with the reporting of the statistical analysis. Could you please address all of their comments carefully?

Please submit your revised manuscript by May 16 2026 11:59PM. If you will need more time than this to complete your revisions, please reply to this message or contact the journal office at globalpubhealth@plos.org. When you're ready to submit your revision, log on to https://www.editorialmanager.com/pgph/ and select the 'Submissions Needing Revision' folder to locate your manuscript file.

Please include the following items when submitting your revised manuscript:

- A letter that responds to each point raised by the editor and reviewer(s). You should upload this letter as a separate file labeled 'Response to Reviewers'.
- A marked-up copy of your manuscript that highlights changes made to the original version. You should upload this as a separate file labeled 'Revised Manuscript with Track Changes'.
- An unmarked version of your revised paper without tracked changes. You should upload this as a separate file labeled 'Manuscript'.

Guidelines for resubmitting your figure files are available below the reviewer comments at the end of this letter.

As the corresponding author, your ORCID iD is verified in the submission system and will appear in the published article. PLOS supports the use of ORCID, and we encourage all coauthors to register for an ORCID iD and use it as well. Please encourage your coauthors to verify their ORCID iD within the submission system before final acceptance, as unverified ORCID iDs will not appear in the published article. *Only*  the individual author can complete the verification step; PLOS staff *cannot*  verify ORCID iDs on behalf of authors.

We look forward to receiving your revised manuscript.

Kind regards,

Alejandro Torrado Pacheco, PhD

PLOS Editor

PLOS Global Public Health

Journal Requirements:

If the reviewer comments include a recommendation to cite specific previously published works, please review and evaluate these publications to determine whether they are relevant and should be cited. There is no requirement to cite these works unless the editor has indicated otherwise.

Additional Editor Comments (if provided):

[Note: HTML markup is below. Please do not edit.]

Reviewers' comments:

Reviewer's Responses to Questions

**Comments to the Author**

1. If the authors have adequately addressed your comments raised in a previous round of review and you feel that this manuscript is now acceptable for publication, you may indicate that here to bypass the “Comments to the Author” section, enter your conflict of interest statement in the “Confidential to Editor” section, and submit your "Accept" recommendation.

Reviewer #6: All comments have been addressed

Reviewer #7: (No Response)

**********

2. Does this manuscript meet PLOS Global Public Health’s publication criteria? Is the manuscript technically sound, and do the data support the conclusions? The manuscript must describe methodologically and ethically rigorous research with conclusions that are appropriately drawn based on the data presented.

Reviewer #6: (No Response)

Reviewer #7: No

**********

3. Has the statistical analysis been performed appropriately and rigorously?

Reviewer #6: Yes

Reviewer #7: No

**********

4. Have the authors made all data underlying the findings in their manuscript fully available (please refer to the Data Availability Statement at the start of the manuscript PDF file)?

The PLOS Data policy requires authors to make all data underlying the findings described in their manuscript fully available without restriction, with rare exception. The data should be provided as part of the manuscript or its supporting information, or deposited to a public repository. For example, in addition to summary statistics, the data points behind means, medians and variance measures should be available. If there are restrictions on publicly sharing data—e.g. participant privacy or use of data from a third party—those must be specified.

Reviewer #6: Yes

Reviewer #7: No

**********

5. Is the manuscript presented in an intelligible fashion and written in standard English?

PLOS Global Public Health does not copyedit accepted manuscripts, so the language in submitted articles must be clear, correct, and unambiguous. Any typographical or grammatical errors should be corrected at revision, so please note any specific errors here.

Reviewer #6: Yes

Reviewer #7: No

**********

6. Review Comments to the Author

Please use the space provided to explain your answers to the questions above. You may also include additional comments for the author, including concerns about dual publication, research ethics, or publication ethics. (Please upload your review as an attachment if it exceeds 20,000 characters)

Reviewer #6: I am satisfied that my concerns have been addressed.

Reviewer #7: The investigators conducted a cross-sectional survey involving patients who attended nine psychiatric hospitals in war torn Eastern DRC between 15th August and 15th December 2021.

Previous reviews of the manuscript seem to focus on the needed procedural clarification and clarity of some explanations and possible connectiveness of the psychiatric , religious, supernatural and medical issues. The statistical clarity overall is lacking despite the section on the Data processing and analysis plan. What about the ordering of type of contact if the numbers are sufficient to do so? See page 5. This is not really explained in the appendix. I can’t open the ‘sav’ file if the pathway is any help.

The analysis is not clearly presented as well. They used a hierarchical separate binary logistic regression model by fitting for each contact stage, to identify factors associated with the first, second, third, and fourth point of contact, all dichotomized into the two categories (PHC system and non-PHC system), recognizing first contact motivations may differ from those guiding later decisions. This is totally confusing and needs expansion. If this is Table 2 (Predictors of seeking mental health services in non-primary health care facilities) then the affect of the ordering is certainly not clear for either the PHC or NPHC data.

Also are the numbers sufficient for the variables or confounders considered in the models. What exactly is the content of the predictors in the models? The limitations in the ‘Discussion’ may need expansion to explain this. The entire statistical presentation needs a thorough edit for clarity.

**********

7. PLOS authors have the option to publish the peer review history of their article (what does this mean?). If published, this will include your full peer review and any attached files.

**Do you want your identity to be public for this peer review?**  If you choose “no”, your identity will remain anonymous but your review may still be made public.

For information about this choice, including consent withdrawal, please see our Privacy Policy.

Reviewer #6: **Yes:**  David Eagle

Reviewer #7: No

**********

[NOTE: If reviewer comments were submitted as an attachment file, they will be attached to this email and accessible via the submission site. Please log into your account, locate the manuscript record, and check for the action link "View Attachments". If this link does not appear, there are no attachment files.]

-->Figure Resubmissions:

While revising your submission, we strongly recommend that you use PLOS’s NAAS tool (https://ngplosjournals.pagemajik.ai/artanalysis) to test your figure files. NAAS can convert your figure files to the TIFF file type and meet basic requirements (such as print size, resolution), or provide you with a report on issues that do not meet our requirements and that NAAS cannot fix.

After uploading your figures to PLOS’s NAAS tool - https://ngplosjournals.pagemajik.ai/artanalysis, NAAS will process the files provided and display the results in the "Uploaded Files" section of the page as the processing is complete. If the uploaded figures meet our requirements (or NAAS is able to fix the files to meet our requirements), the figure will be marked as "fixed" above. If NAAS is unable to fix the files, a red "failed" label will appear above. When NAAS has confirmed that the figure files meet our requirements, please download the file via the download option, and include these NAAS processed figure files when submitting your revised manuscript.

# Author comment: Author response to Decision Letter 3

**DOI:** 10.1371/journal.pgph.0006715.r006

16 May 2026

Attachment Submitted filename: Responses to reviewers 2.pdf

# Peer review: Decision Letter 3

**DOI:** 10.1371/journal.pgph.0006715.r007

**Authors:** Collins Asweto, Helen Howard, Alejandro Torrado Pacheco, Julia Robinson

5 Jun 2026

Beliefs, Referrals, and Mental Healthcare Pathways in the Eastern Democratic Republic of Congo

PGPH-D-25-01441R3

Dear Dr Mutume Nzanzu Vivalya,

We are pleased to inform you that your manuscript 'Beliefs, Referrals, and Mental Healthcare Pathways in the Eastern Democratic Republic of Congo ' has been provisionally accepted for publication in PLOS Global Public Health.

Before your manuscript can be formally accepted you will need to complete some formatting changes, which you will receive in a follow up email. A member of our team will be in touch with a set of requests.

Please note that your manuscript will not be scheduled for publication until you have made the required changes, so a swift response is appreciated.

IMPORTANT: The editorial review process is now complete. PLOS will only permit corrections to spelling, formatting or significant scientific errors from this point onwards. Requests for major changes, or any which affect the scientific understanding of your work, will cause delays to the publication date of your manuscript.

If your institution or institutions have a press office, please notify them about your upcoming paper to help maximize its impact. If they'll be preparing press materials, please inform our press team as soon as possible -- no later than 48 hours after receiving the formal acceptance. Your manuscript will remain under strict press embargo until 2 pm Eastern Time on the date of publication. For more information, please contact globalpubhealth@plos.org.

Thank you again for supporting Open Access publishing; we are looking forward to publishing your work in PLOS Global Public Health.

Best regards,

Julia Robinson

Executive Editor

PLOS Global Public Health

***********************************************************

Reviewer Comments (if any, and for reference):

Reviewer's Responses to Questions

**Comments to the Author**

1. If the authors have adequately addressed your comments raised in a previous round of review and you feel that this manuscript is now acceptable for publication, you may indicate that here to bypass the “Comments to the Author” section, enter your conflict of interest statement in the “Confidential to Editor” section, and submit your "Accept" recommendation.

Reviewer #7: All comments have been addressed

**********

2. Does this manuscript meet PLOS Global Public Health’s publication criteria? Is the manuscript technically sound, and do the data support the conclusions? The manuscript must describe methodologically and ethically rigorous research with conclusions that are appropriately drawn based on the data presented.

Reviewer #7: Yes

**********

3. Has the statistical analysis been performed appropriately and rigorously?

Reviewer #7: Yes

**********

4. Have the authors made all data underlying the findings in their manuscript fully available (please refer to the Data Availability Statement at the start of the manuscript PDF file)?

The PLOS Data policy requires authors to make all data underlying the findings described in their manuscript fully available without restriction, with rare exception. The data should be provided as part of the manuscript or its supporting information, or deposited to a public repository. For example, in addition to summary statistics, the data points behind means, medians and variance measures should be available. If there are restrictions on publicly sharing data—e.g. participant privacy or use of data from a third party—those must be specified.

Reviewer #7: Yes

**********

5. Is the manuscript presented in an intelligible fashion and written in standard English?

PLOS Global Public Health does not copyedit accepted manuscripts, so the language in submitted articles must be clear, correct, and unambiguous. Any typographical or grammatical errors should be corrected at revision, so please note any specific errors here.

Reviewer #7: Yes

**********

6. Review Comments to the Author

Please use the space provided to explain your answers to the questions above. You may also include additional comments for the author, including concerns about dual publication, research ethics, or publication ethics. (Please upload your review as an attachment if it exceeds 20,000 characters)

Reviewer #7: All comments of a descriptive nature have been met.

**********

7. PLOS authors have the option to publish the peer review history of their article (what does this mean?). If published, this will include your full peer review and any attached files.

**Do you want your identity to be public for this peer review?**  If you choose “no”, your identity will remain anonymous but your review may still be made public.

For information about this choice, including consent withdrawal, please see our Privacy Policy.

Reviewer #7: No

**********
